# Genome-wide association study of *Verticillium longisporum* resistance in *Brassica* genotypes

**DOI:** 10.3389/fpls.2024.1436982

**Published:** 2024-08-27

**Authors:** Yixiao Wang, Rudolph Fredua-Agyeman, Zhiyu Yu, Sheau-Fang Hwang, Stephen E. Strelkov

**Affiliations:** Department of Agricultural, Food and Nutritional Science, University of Alberta, Edmonton, AB, Canada

**Keywords:** *Brassica*, genomic hotspots, genotypes, markers, resistance, *Verticillium longisporum*, Verticillium stripe

## Abstract

Verticillium stripe, caused by *Verticillium longisporum*, presents an emerging threat to Canadian canola (*Brassica napus*). Initially detected in Manitoba in 2014, the presence of this pathogen has since been confirmed across western Canada. Infections by *V. longisporum* can result in yield losses of up to 50%, which is a cause for concern given the susceptibility of most commercial Canadian canola cultivars. The objective of this study was to screen a collection of 211 *Brassica* genotypes for their reactions to *V. longisporum*, and to use genome-wide association study (GWAS) to identify single nucleotide polymorphism (SNP) markers for resistance. The plant material consisted of 110 rutabaga (*B. napus* ssp. *napobrassica*), 35 canola, 40 *Brassica rapa*, and 15 *Brassica oleracea* accessions or cultivars, alongside 11 hosts of the European Clubroot Differential (ECD) set. These materials were screened for resistance under greenhouse conditions and were genotyped using a 19K *Brassica* SNP array. Three general linear models (GLM), four mixed linear models (MLM), and three GWAS methods were employed to evaluate the markers. Eleven non-commercial *Brassica* accessions and 9 out of 35 commercial canola cultivars displayed a low normalized area under the disease progress curve (AUDPC_norm._). The non-commercial accessions could prove valuable as potential sources of resistance against *V. longisporum*. Forty-five SNP markers were identified to be significantly associated with *V. longisporum* resistance using single-SNP based GWAS analysis. In comparison, haplotype-based GWAS analyses identified 10 to 25 haplotype blocks to be significantly associated with *V. longisporum* resistance. Between 20% and 56% of QTLs identified by the more conventional single-SNP based GWAS analysis were also detected by the haplotype-based GWAS analysis. The overlapping genomic regions identified by the two GWAS methods present promising hotspots for marker-assisted selection in the future development of Verticillium stripe-resistant canola.

## Introduction

Verticillium stripe, caused by the fungal pathogen *Verticillium longisporum* (C. Stark) Karapapa, Bainbridge and Heale, is an important soilborne disease of canola (*Brassica napus* L.) in Canada. The first case of Verticillium stripe in this country was identified in a canola field in Manitoba in 2014 (Canadian Food Inspection Agency, 2018). Subsequently, *V. longisporum* has been detected in other Canadian provinces, including British Columbia, Alberta, Saskatchewan, Ontario, and Quebec (Canadian Food Inspection Agency, 2018). Yield losses due to *V. longisporum* infection were reported to range from approximately 10% to 50% on canola, although they could exceed 80% on a single plant (Dunker et al., 2008). Since the survival structures (microsclerotia) of *V. longisporum* can persist in the soil for up to 10 years (Schnathorst, 1981), strategies such as minimizing soil movement, implementing longer rotations out of host crops, and good weed management can potentially reduce *V. longisporum* inoculum levels (Johansson et al., 2006). However, these strategies may not be practical for growers due to economic concerns. Moreover, there are currently no registered fungicides available for controlling this disease (Dunker et al., 2008). Therefore, genetic resistance stands out as the most effective and environmentally friendly approach for managing Verticillium stripe. Unfortunately, no commercial canola varieties in Canada have been registered as resistant to *V. longisporum* (Norman, 2023).

*Verticillium longisporum* mainly attacks hosts in the *Brassicaceae* family, such as *B. napus* (canola/oilseed rape and rutabaga), *Brassica rapa* L. (including pak choy, Chinese cabbage, and turnip), *Brassica oleracea* L. (including broccoli, cauliflower, cabbage, and kale), and *Brassica juncea* L. (including brown and leaf mustard) (Zeise and Von Tiedemann, 2002; Depotter et al., 2016). Some progenitor species of *B. napus* (AACC, *n* = 19), including *B. rapa* (AA, *n* = 10) and *B. oleracea* (CC, *n* = 9) (Nagaharu, 1935), have been reported to exhibit higher levels of resistance to several significant canola diseases such as blackleg (Zou and Fernando, 2024), clubroot (Fredua-Agyeman et al., 2020), and Sclerotinia stem rot (Khan et al., 2023). Consequently, the screening of various *Brassica* species for genetic resistance to *V. longisporum* is an important breeding objective. Rygulla et al. (2007) identified two major quantitative trait loci (QTL) for *V. longisporum* resistance on chromosomes C04 and C05. Additionally, Obermeier et al. (2013) found a major QTL on chromosome C05 and a minor QTL on C01, both correlated with *V. longisporum* resistance, whereas Gabur et al. (2020) reported a QTL for resistance on chromosome C09. In a recent study, Su et al. (2023) demonstrated that the MYB transcription factor BrMYB108 in *B. rapa* directly targets genes encoding respiratory burst oxidase homologues, leading to resistance against *V. longisporum* through the regulation of reactive oxygen species (ROS) generation (Su et al., 2023).

However, to the best of our knowledge, no screening or resistance gene/QTL detection studies have been conducted in Canada for the identification of *Brassica* germplasm suitable for breeding *V. longisporum* resistance in commercial canola cultivars. Therefore, the objective of this study was to screen a large collection of rutabaga (*B. napus* ssp. *napobrassica*) accessions and commercial canola cultivars from Canada, as well as *B. rapa* and *B. oleracea* genotypes from China, for resistance to this fungus. Additionally, a genome-wide association study (GWAS) was utilized to identify accessions and genomic regions associated with *V. longisporum* resistance.

## Materials and methods

### Plant materials

Two-hundred eleven *Brassica* accessions, commercial cultivars, and differential hosts were evaluated for their reaction to *V. longisporum*. Among these were 110 rutabaga (*B. napus* spp. *napobrassica*) accessions previously screened for clubroot resistance by Fredua-Agyeman et al. (2020), and utilized in genetic diversity studies by Yu et al. (2021). In addition, the evaluation included 35 Canadian canola cultivars, 40 *B. rapa* vegetable cultivars, and 15 *B. oleracea* vegetable cultivars from China. Furthermore, 11 hosts of the European Clubroot Differential set (ECD; Buczacki et al., 1975) were tested, including ECD 06, ECD 08, ECD 09, ECD 10 (*B. napus*), ECD 01, ECD 02, ECD 03, ECD 04, ECD 05 (‘Granaat’) (*B. rapa*), ECD 11, and ECD 13 (*B. oleracea*). Among these 211 *Brassica* genotypes, the Canadian canola cultivar ‘Westar’ was included as a susceptible check, while *B. rapa* var. *pekinensis* ‘Granaat’ (ECD 05) served as a moderately resistant check (Rygulla et al., 2007). The details of the plant materials used are presented in **Supplementary Table S1**.

### Resistance phenotyping

The single-spore isolate VL 43 of *V. longisporum*, collected from an infected canola plant sampled near Edmonton, Alberta (Cui et al., 2022), was cultured in Petri dishes (9-cm diameter) filled with potato dextrose agar (PDA). The multiplex PCR method described by Inderbitzin et al. (2013) was employed to identify isolate VL 43 as *V. longisporum* lineage A1/D1. Cultures were incubated in darkness at room temperature for 28 days before harvesting the conidia. Briefly, 10 mL of sterile distilled water was added to each Petri dish, and a sterile inoculating loop was used to gently dislodge the spores. The resulting conidial suspension was filtered through four layers of sterile cheesecloth to remove mycelial fragments. The spore concentration was then estimated using a haemocytometer (Hausser Scientific, Horsham, Pennsylvania, USA), and adjusted to 1 × 10^6^ spores mL^−1^ with sterile distilled water.

Seven-day old seedlings of the 211 *Brassica* genotypes were inoculated using the root-dip method as described by Cui et al. (2022). Non-inoculated controls were dipped in sterile distilled water instead. The experimental setup consisted of 32 L plastic tubs filled with Sunshine Mix #4 growing mix (Sun Gro Horticulture, Vilna, Alberta, Canada). Each tub accommodated five seedlings of the same genotype, with four *Brassica* genotypes per tub, totaling 20 plants (5 plants × 4 genotypes) per tub and each genotype had 4 replicates. The plants were maintained in a greenhouse under an 18-h photoperiod (22°C day/16°C night).

Disease severity assessments were conducted weekly for each plant over a 4-week period. The assessment utilized a 1–9 rating scale as described by Eynck et al. (2009), where a rating of 1 = no symptoms, while 9 = the plant is dead. The experiment was arranged in a randomized completely block design with four replicates, and was independently repeated.

### Statistical analysis of the disease data

The area under the disease progress curve (AUDPC) was calculated for each host genotype based on Verticillium stripe severity using the formula described by Campbell and Madden (1990): AUDPC = $\sum_{i=1}^{n}({\text{y}}_{\text{i}+}{\text{y}}_{\text{i}+1}/2)\times ({\text{t}}_{\text{i}+1}-{\text{ t}}_{\text{i}})$ , where $y_{\text{i}}$ is the disease severity for each observation number i, ${\text{t}}_{\text{i}}$ is the number of days after inoculation at the time of observation number i, and *n* is the number of observations. Non-inoculated plants were also assessed on the same scale at the same times. A net AUDPC value (AUDPC_net_) was calculated following Eynck et al. (2009): AUDPC_net_ = AUDPC(X_inoc._) – AUDPC(X_contr._), where X_inoc._ is the inoculated plants and X_contr._ is non-inoculated controls.

The AUDPC values were normalized for each genotype relative to the susceptible check ‘Westar’ and moderately resistant check ‘Granaat’ to account for fluctuating disease severity between trials. The normalized AUDPC (AUDPC_norm._) was calculated according to Eynck et al. (2009):


$$\text{AUDPCnorm}.=\frac{\text{AUDPC}}{(\text{AUDPC Westar}+\text{AUDPC Granaat})/2}$$

The phenotype data was analyzed statistically using R: A Language and Environment for Statistical Computing (R Core Team, 2013). *Brassica* genotypes with significantly lower AUDPC_norm._ (*P* ≤ 0.05) compared to the moderately resistant cultivar ‘Granaat’ were considered resistant (Rygulla et al., 2007; Eynck et al., 2009). If 0.05 < *P* ≤ 0.1, the genotypes were regarded as moderately resistant. In addition, for those not assigned as resistant or moderately resistant, the susceptible check ‘Westar’ was also used for comparison. *Brassica* genotypes with *P* ≤ 0.05 were regarded as susceptible and *P >* 0.05 were considered as moderately susceptible.

### SNP genotyping

SNP genotyping was performed on all 211 *Brassica* genotypes using a *Brassica* 19K SNP array from TraitGenetics GmbH (Gatersleben. Germany). This array included 9,966 SNP markers on the A-genome, 7,740 SNP markers on the C-genome, and 1,146 SNP markers on scaffolds. After filtering monomorphic, low-coverage site markers, as well as markers with minor allele frequency (MAF) ≤ 0.05 and those missing data for >10%, 4,972 A-genome markers and 4,621 C-genome markers were retained for the GWAS. The GWAS was conducted separately on the 45 *B. rapa* (AA) and 149 *B. napus* (AACC) accessions using the A-genome markers, and on the 17 *B. oleracea* (CC) and 149 *B. napus* accessions using the C-genome markers. Additionally, the average inter-SNP marker distance was determined for each combination and each chromosome (**Table 1**).

**Table 1:** Single nucleotide polymorphism (SNP) marker density and extent of intrachromosomal linkage disequilibrium (LD) in *Brassica rapa*, *Brassica napus* and *Brassica oleracea* genotypes included in a genome-wide association study of resistance to *Verticillium longisporum*.

Linkage group or Chromosome	Total # of SNP markers	# Filtered SNP markers	Length covered (kb)	Average inter-SNP marker distance (kb)	Pairwise comparisons of all linked SNP markers	Number (%) of SNP pairs in significant LD*	Average r^2^ value/chromosome	Estimated LD decay (Mb) ^ϕ^
A01	865	430	29050.96	67.7	92235	24630 (26.7%)	0.153	0.58
A02	787	427	29805.34	70.0	90951	28445 (31.3%)	0.178	0.91
A03	1553	779	37644.04	48.4	303031	71089 (23.5%)	0.148	0.48
A04	984	493	22049.36	44.8	121278	31756 (26.2%)	0.161	0.58
A05	981	455	29222.52	64.4	103285	29260 (28.3%)	0.167	0.75
A06	1102	530	31805.48	60.1	140185	42146 (30.1%)	0.157	0.61
A07	1395	667	27493.45	41.3	222111	52218 (23.5%)	0.148	0.43
A08	673	382	21796.85	57.2	72771	27800 (38.2%)	0.223	0.42
A09	811	370	42688.79	115.7	68265	17568 (25.7%)	0.144	1.15
A10	815	439	20088.28	45.9	96141	30124 (31.3%)	0.167	0.44
A-genome	9966	4972	291646.1	61.6 ± 21.5	1310253	355036 (27.1%)	0.163	0.60
C01	790	311	43826.6	141.4	48205	20992 (43.5%)	0.358	0.99
C02	813	528	61056.6	115.9	139128	49779 (35.8%)	0.280	1.10
C03	1524	829	61857.1	74.7	343206	119219 (34.7%)	0.282	0.81
C04	1147	798	56008.8	70.3	318003	105924 (33.3%)	0.267	0.65
C05	558	329	46342.5	141.3	53956	21564 (40.0%)	0.277	1.45
C06	816	545	45790.4	84.2	148240	46786 (31.6%)	0.255	0.82
C07	871	587	38104.3	65.0	171991	69196(40.2%)	0.284	0.68
C08	734	463	51664.0	111.8	106953	41807 (39.1%)	0.243	0.80
C09	487	231	58767.0	255.5	26565	10633 (40.0%)	0.265	1.85
C-genome	7740	4621	463417.2	117.8 ± 59.3	1356247	485900 (35.8%)	0.277	0.42

^*^The number and percentages of SNP pairs in significant LD were determined from Chi-squared tests at p-value < 0.001. ^ϕ^The extent of LD decay was estimated from the projection of the intersection between the fitted curve of the data points and the 95th percentile of an unlinked r^2^ threshold line (background LD) onto the physical distance axis.

### Linkage disequilibrium estimation

Intra-chromosomal linkage disequilibrium (LD) between allelic values at two loci was estimated using Pearson’s squared correlation coefficient (r^2^) statistic with TASSEL 5.0 (Bradbury et al., 2007). To determine the significance of pairwise marker r^2^-values, *P* < 0.001 of the Chi-square (χ2) statistic for each SNP pair was used according to Fredua-Agyeman et al. (2020). The LD decay curves were determined by calculating the Chi-square (χ^2^) statistic for each SNP pair in relation to physical map distance (in Mb) using R v. 4.3.2 (R Core Team, 2013). The extent of LD was estimated based on the interaction of the fitted LD decay curve and r^2^-threshold lines for each chromosome (Breseghello and Sorrells, 2006; Bellucci et al., 2015).

### Population structure analysis

To determine the population structure (Θ) of the *Brassica* accessions used in this study, a Bayesian clustering approach was employed. Burn-in periods ranged from 5,000 to 100,000 iterations, and Markov Chain Monte Carlo (MCMC) analyses ranged from 5,000 to 100,000 permutations through the population-genetic software *STRUCTURE* v. 2.3.4 (Pritchard et al., 2000). The *Brassica rapa* + *B. napus* genotypes and *B. oleracea* + *B. napus* genotypes were analyzed separately to determine the number of genetically homogeneous clusters (*K*) based on 4,972 and 4,621 SNP markers, respectively. Runs for each *K*=1–10 were replicated 10 times. The number of clusters and average log-likelihood plots were determined according to Evanno et al. (2005) through STRUCTURE HARVESTER (Earl and vonHoldt, 2012).

The genetic diversity of *B. rapa* + *B. napus* genotypes and *B. oleracea* + *B. napus* genotypes was determined separately. This analysis was based on 4,972 A-genome markers and 4,621 C-genome markers. The unweighted pair group method with arithmetic mean (UPGMA) and the neighbor joining (NJ) method implemented in TASSEL v. 5.0 were used to generate phylogenetic trees.

### SNP-based genome-wide association analyses

Three general linear models (GLM) and four mixed linear models (MLM) implemented in TASSEL v. 5.0 (Bradbury et al., 2007) were tested for the SNP-based marker-trait association studies. The GLM tested consisted of the population structure (Q)-only, Kinship (K)-only, and Principal Coordinate Analysis (PCA)-only models. The MLM models comprised Q+K, PCA+K, Q+PCA and PCA+D (Distance matrices) (Fredua-Agyeman et al., 2020). Furthermore, three additional GWAS methods were employed using the GAPIT v. 3 (Wang and Zhang, 2021) package in R. These included the Bayesian-information and Linkage-disequilibrium Iteratively Nested Keyway (BLINK) (Huang et al., 2019), the Fixed and random model Circulating Probability Unification (FarmCPU) (Liu et al., 2016), and the Multiple Locus Mixed Linear Model (MLMM) (Segura et al., 2012).

The SNP-based GWAS was conducted for the *B. rapa* + *B. napus* accessions using the 4,972 A-genome SNP marker data. Two independent AUDPC measurements and the average of two sets of AUDPC phenotype data were utilized. This analysis was performed using the seven models and three methods noted above. Similarly, the 4,621 C-genome SNP marker data and two independent measurements of AUDPC, along with the average of two sets of AUDPC data for each genotype, were used for the GWAS of the *B. oleracea* + *B. napus* genotypes. For each model/method and genotype combination, Quantile-Quantile (Q-Q) plots were examined to identify which plot showed the least amount of deviation from the expected –log_10_ *P-*value. Significant markers associated with Verticillium stripe resistance were identified by examining the best-fitted Q-Q plots and Manhattan plots. These plots were generated using the CMplot package in R. To establish the significance cut-off (-log_10_ (0.05/n), n = number of markers), the Bonferroni correction was applied (Benjamini and Hochberg, 1995). A slightly lower threshold of -log_10_ *P* = 3.0 was employed for association. Significant SNP markers associated with Verticillium stripe resistance were identified using the various models and methods.

### Construction of haplotype blocks

Haplotype association tests were performed using three different algorithms (Confidence Interval [CI], Four Gamete Rule [FGR], and Solid Spine of LD [SS]) implemented in the software Haploview v 4.2 (Barrett et al., 2005). Haplotype blocks were constructed for each chromosome separately to identify SNPs in the same haploblock and to investigate the combined effect of the linked significant SNPs. Each analysis was carried out using the default settings and the standardized disequilibrium coefficient (D’) was used to determine the LD between SNP markers and to generate halpoview LD plots. Blocks were formed if ≥ 95% of the comparisons exhibited strong LD. The haplotype blocks were transformed into multiallelic markers and used for haplotype-trait association analyses (Abed and Belzile, 2019; Bajgain and Anderson, 2021).

### Candidate gene prediction

The probe sequences of SNP markers significantly associated with VL resistance were utilized in BlastN searches of the *B. rapa* (AA) genome assembly CAAS_Brap_v3.01, *B. oleracea* (CC) genome assembly BOL, and *B. napus* (AACC) genome assembly Da-Ae in the EnsemblPlants (plants.ensembl.org) and National Centre for Biotechnology Information (www.ncbi.nlm.nih.gov) databases. Using a threshold of ≥ 90% identity and an E-value ≤ 1e^-20^, candidate genes were mapped to the reference genomes. The physical locations of these genes were determined based on the significant SNPs and haploblocks in strong LD.

## Results

### Verticillium stripe phenotyping

**Figure 1:**
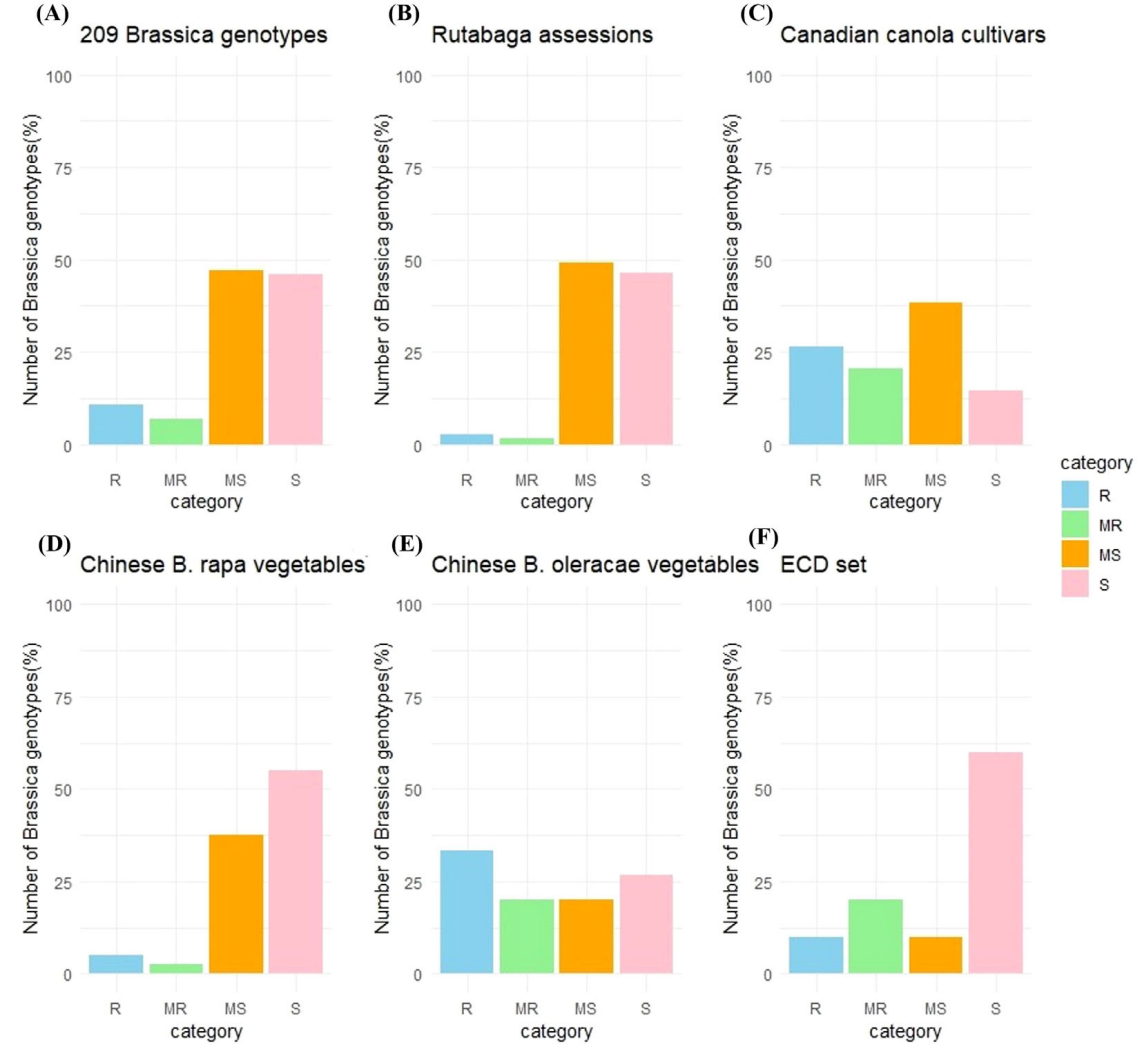
Reaction of *Brassica* genotypes to inoculation with *Verticillium longisporum*. Genotypes were rated as susceptible (S), moderately susceptible (MS), moderately resistant (MR), or resistant (R) to the fungus based on disease severity. Frequency distributions for host reactions are shown for **(A)** the entire collection of 209 genotypes excluding the susceptible and moderately resistant checks; **(B)** 110 rutabagas (*B. napus* ssp. *napobrassica*); **(C)** 34 canola (*B. napus*) cultivars; **(D)** 39 vegetable-type *B. rapa*; **(E)** 15 vegetable-type *B. oleracea*; and **(F)** 10 selected hosts of the European Clubroot Differential (ECD) set, excluding the moderately resistant-check ECD 05.

Excluding the susceptible and moderately resistant checks, 20 (9.6%) of the remaining 209 *Brassica* genotypes tested were classified as resistant (R), 13 (6.2%) as moderately resistant (MR), 89 (42.6%) as moderately susceptible (MS), and 87 (41.6%) as susceptible (S) (**Figure 1A**).

Among the 110 rutabaga accessions screened, three (2.7%) were R, two (1.8%) were MR, 54 (49.1%) were MS, and 51 (46.4%) were S (**Figure 1B**). The AUDPC_norm._ scores from the first and second rounds of screening ranged from 0.295 ± 0.181 to 2.741 ± 0.717, and from 0.242 ± 0.095 to 4.497 ± 2.051, respectively, while the average AUDPC_norm._ score of the two rounds ranged from 0.269 ± 0.137 to 3.533 ± 1.690 (**Supplementary Figure S1A**).

The AUDPC_norm._ values for 34 of the 35 Canadian commercial canola cultivars, excluding the susceptible check ‘Westar’, showed that nine (26.5%) were classified as R, seven (14.7%) as MR, 13 (47.1%) as MS, and five (11.8%) as S (**Figure 1C**). The first round of AUDPC_norm._ scores ranged from 0.016 ± 0.028 to 3.448 ± 0.442, the second round ranged from 0.177 ± 0.037 to 2.541 ± 0.836, and the average AUDPC_norm._ scores from the two rounds ranged from 0.101 ± 0.188 to 2.994 ± 0.786 (**Supplementary Figure S1B**).

Among the 40 *B. rapa* vegetable cultivars from China, two (5.0%) were classified as R, one (2.5%) as MR, 15 (37.5%) as MS, and 22 (55.0%) as S (**Figure 1D**). The AUDPC_norm._ scores from the first and second rounds ranged from 0.120 ± 0.034 to 3.370 ± 0.012, and from 0.138 ± 0.037 to 3.040 ± 0.421, respectively, while the average AUDPC_norm._ scores from the two rounds ranged from 0.129 ± 0.034 to 3.202 ± 0.084 (**Supplementary Figure S1C**).

In the case of the 15 *B. oleracea* vegetable cultivars from China, five (33.3%) were classified as R, three (20.0%) as MR, three (20.0%) as MS, and four (26.7%) as S (**Figure 1E**). The first round of AUDPC_norm._ scores ranged from 0.158 ± 0.026 to 0.918 ± 0.411, the second round ranged from 0.159 ± 0.063 to 1.718 ± 0.928, and the average AUDPC_norm._ score from the two rounds ranged from 0.168 ± 0.058 to 1.315 ± 0.709 (**Supplementary Figure S1D**).

Besides the moderately resistant check ECD 05 (‘Grannat’), among the other 10 selected hosts of the ECD set, one (10.0%) (ECD 11) was classified as R, two (20.0%) (ECD 08 and ECD13) as MR, one (10.0%) as MS (ECD 09), and six (60.0%) (ECD 01, ECD 02, ECD 03, ECD 04, ECD 06 and ECD10) as susceptible (S) (**Figure 1F**). The AUDPC_norm._ scores from the first and second rounds of screening ranged from 0.064 ± 0.051 to 3.599 ± 0.232, and from 0.208 ± 0.134 to 2.989 ± 0.338, respectively. The average AUDPC_norm._ scores from the two rounds ranged from 0.186 ± 0.161 to 3.294 ± 0.473 (**Supplementary Figure S1E**).

**Figure 2:**
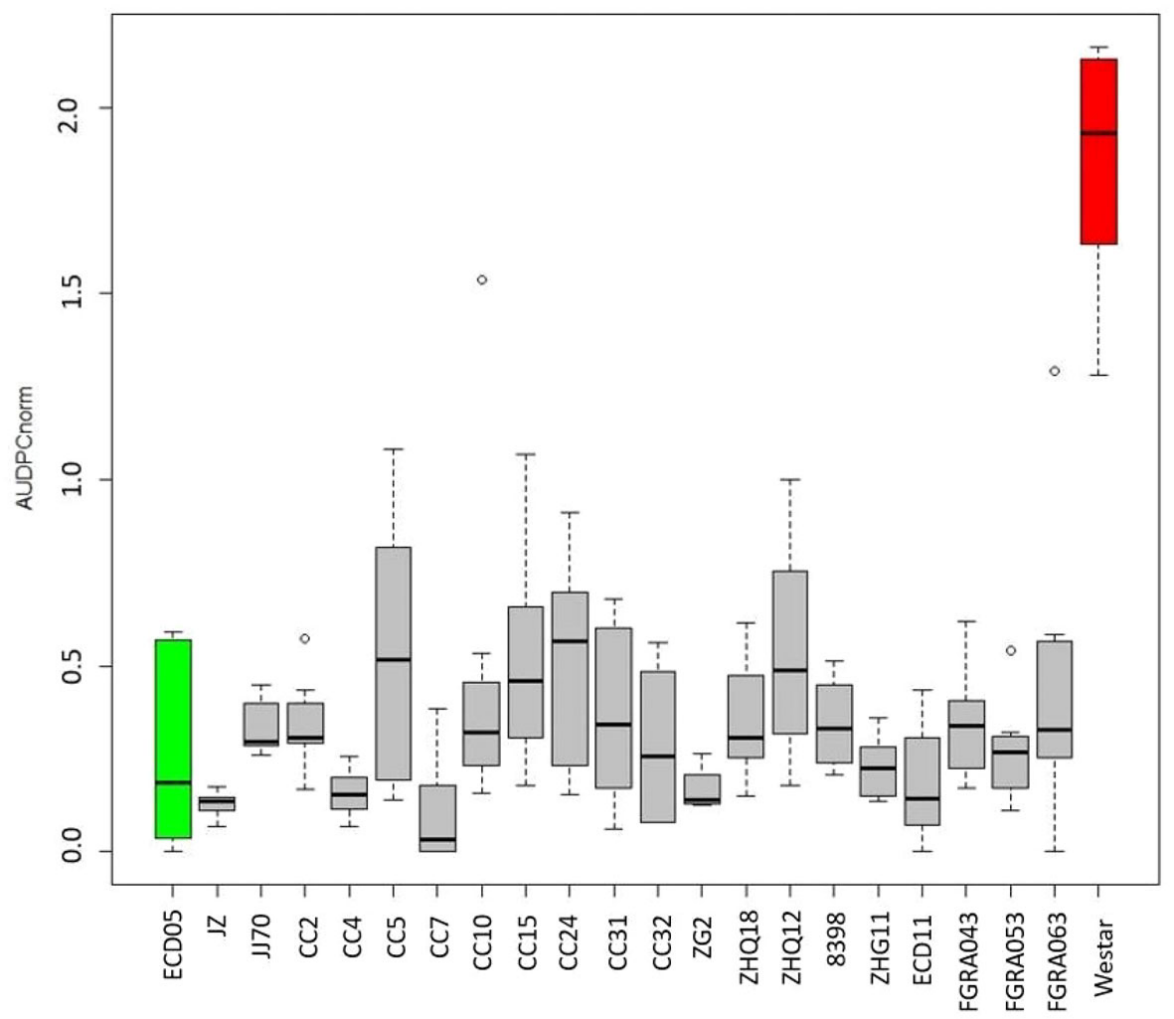
Normalized area under the disease progress curve (AUDPC_norm._) for 20 *Brassica* genotypes showing resistance to *Verticillium longisporum.* The AUDPC_norm._ was calculated from disease severities rated from 1–9 following Eynck et al. (2009), where 1 = no symptoms and 9 = the plant is dead. The grey bars show the range of the maximum and minimum AUDPC_norm._, and the black lines in each box indicate the mean of AUDPC_norm._ values among eight replicates in two independent repeats. The green bar denotes the moderately resistant check *B. rapa* var. *pekinensis* ‘Granaat’ (ECD 05), while the red bar denotes the susceptible check *B. napus* ‘Westar’. The other genotypes shown include the rutabagas (*B. napus* ssp. *napobrassica*) FGRA043, FGRA053, and FGRA063; canola (*B. napus*) cultivars CC2, CC4, CC5, CC7, CC10 and CC15; *B. rapa* cultivars ‘Jingyan Zikuaicai’ (JZ), and ‘Jingjian No.70’ (JJ70); *B. oleracea* cultivars ‘Zigan2’ (ZG2), ‘Zhongqing 18’ (ZHQ18), ‘Zhongqing 12’ (ZHQ12), ‘Zhonggan 11’ (ZHQ11), and ‘8398’; and ECD 11.

Based on the average AUDPC_norm._ scores, 20 *Brassica* genotypes showing a strong level of resistance to *V. longisporum* were identified. These included the rutabagas FGRA043, FGRA053, and FGRA063; canola cultivars CC2, CC4, CC5, CC7, CC10 and CC15; *B. rapa* cultivars ‘Jingyan Zikuaicai’, and ‘Jingjian No.70’; *B. oleracea* cultivars ‘Zigan2’, ‘Zhongqing 18’, ‘Zhongqing 12’, ‘Zhonggan 11’, and ‘8398’; and ECD 11 (**Figure 2**).

### Distribution of polymorphic SNP markers

**Table 1** presents the number and distribution of SNP markers retained in the GWAS to determine resistance to *V. longisporum*. In the GWAS of the *B. rapa* and *B. napus* accessions, the mean number of filtered SNP markers was 497.2 ± 130.6, ranging from 370 on chromosome A09 to 779 on chromosome A03 (**Table 1**). The filtered set of 4,972 markers covered 291.6 Mb of the A-genome in *B. rapa* and *B. napus* (**Table 1**). The mean inter-SNP marker distance or density for the A-genome was 61.6 ± 21.5 Kb, ranging from 41.3 on chromosome A07 to 115.7 on chromosome A09 (**Table 1**). In the GWAS of the *B. oleracea* and *B. napus* accessions, the mean number of filtered SNP markers was 513.4 ± 207.5, ranging from 231 on chromosome C09 to 829 on chromosome C03 (**Table 1**). The filtered set of 4,621 markers covered 463.4 Mb of the C-genome in *B. oleracea* and *B. napus* (**Table 1**). The mean inter-SNP marker distance or density for the C-genome was 117.8 ± 59.3 Kb, ranging from 65.0 on chromosome C07 to chromosome C09 255.5 (**Table 1**).

### Linkage disequilibrium

The average of the squared allele correlation LD (*r* ^2^) for all chromosomes is presented in **Table 1** and the plots of correlation coefficient (*r* ^2^) and physical distance (in Mb) for SNP markers on chromosomes A and C are presented in **Supplementary Figure S2**. The mean r^2^ value for the A-genome of *B. rapa* and *B. napus* was calculated to be 0.163, ranging from 0.144 on chromosome A09 to 0.223 on chromosome A08 (**Table 1**). The average extent of LD decay for the 10 A-genome chromosomes (A_1_-A_10_) ranged from 0.42 Mb on chromosome A08 to 1.15 Mb on chromosome A09, with an estimated mean of 0.60 Mb (**Table 1**). The mean r^2^ for the C-genome of *B. oleracea* and *B. napus* was 0.277, ranging from 0.243 on chromosome C08 to 0.358 on chromosome C01 (**Table 1**). The estimated mean LD decay for the nine C-genome chromosomes (C_1_ to C_9_) ranged from 0.68 Mb on chromosome C07 to 1.85 Mb on chromosome C09, with an estimated mean of 0.42 Mb (**Table 1**). Therefore, the extent of LD for the C-genome chromosomes was slightly greater than for the A-genome chromosomes.

### Population structure analyses

**Figure 3:**
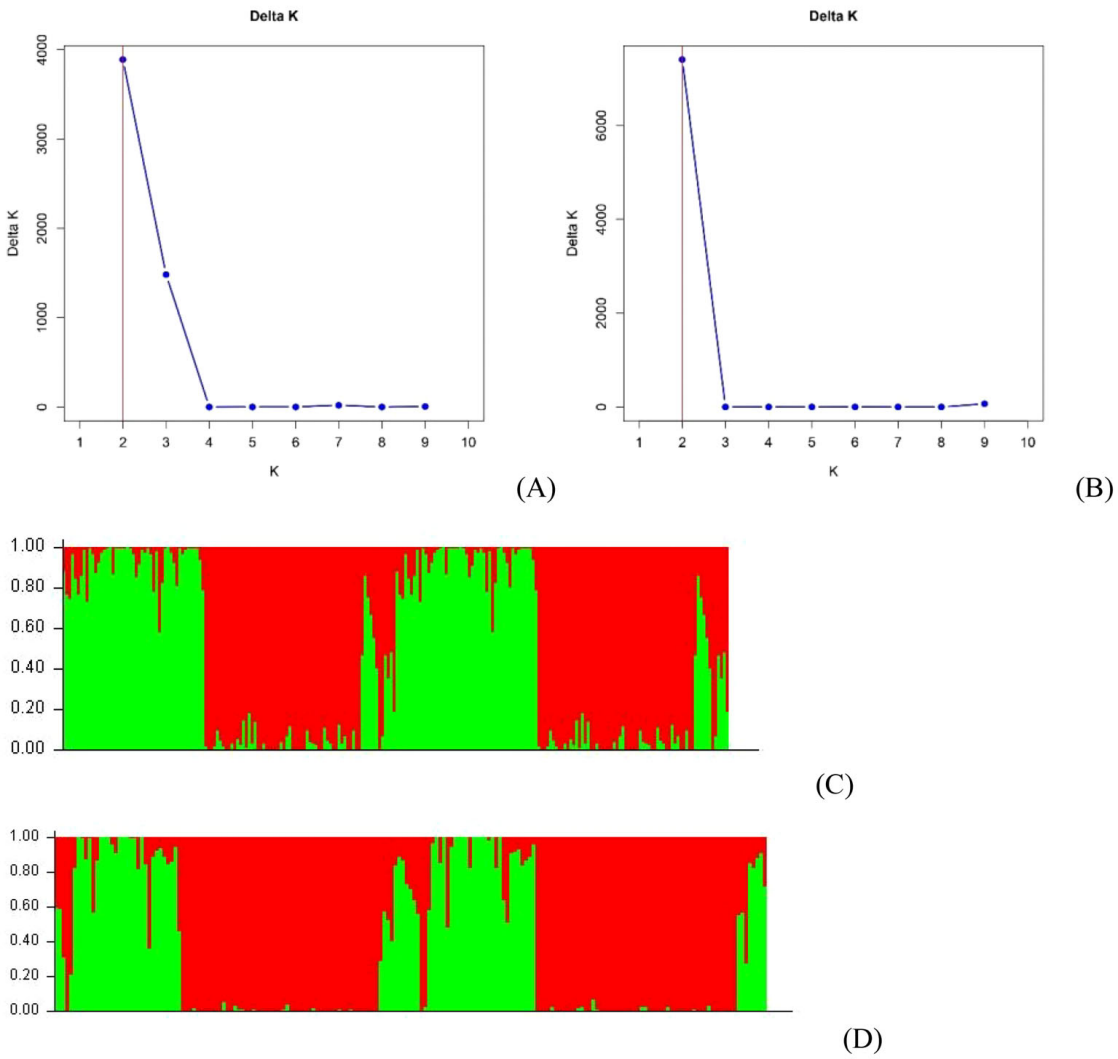
Bayesian cluster analysis of 211 *Brassica* accessions including *B. napus*, *B. oleracea* and *B. rapa* estimated with STRUCTURE using 50,000 burn-in iterations and Markov Chain Monte Carlo (MCMC) lengths. The value of K, determined following Evanno et al. (2005), indicated two clusters for the *B. rapa* and *B. napus* genotypes **(A)**, and for the *B. oleracea* and *B. napus* genotypes **(B)**, in all runs. Detailed Bayesian clustering of the *B. rapa* and *B. napus* genotypes **(C)**, and of the *B. oleracea* and *B. napus* genotypes **(D)**, is also shown, with each color representing one ancestry component. The simplified view suggests two ancestral populations.

Two clusters (*K*=2) were determined at all runs (10,000, 50,000 and 100,000 burn-in iterations and MCMC lengths) by the method of Evanno et al. (2005) using *STRUCTURE* for the analyses of both *B. rapa* + *B. napus* (**Figure 3A**) and *B. napus* + *B. oleracea* (**Figure 3B**). At a probability of 70%, 77 (39.7%) of the *B. rapa* and *B. napus* genotypes were placed in group 1, 108 (55.7%) were placed in group 2, while 9 (4.6%) were classified as admixtures (**Figure 3C**). In group 1, there were 45 *B. rapa* genotypes, including 40 vegetable cultivars from China and the ECD lines 01–05, along with 32 Canadian canola cultivars. In Group 2, there were 106 rutabagas, along with ECD 10 and one Canadian canola cultivar. Additionally, there were two Canadian canola cultivars, along with ECD 06–09 and four rutabagas classified as admixtures.

Among the *B. oleracea* and *B. napus* genotypes, 51 (30.7%) were placed in group 1, 110 (66.3%) in group 2, while five (3.0%) were classified as admixtures based on a probability of 70% (**Figure 3D**). In group 1, there were 17 *B. oleracea* genotypes, including 15 vegetable cultivars, ECD 11, and ECD 13, as well as 34 Canadian canola cultivars. Group 2 consisted of 108 rutabagas, ECD 10, and one Canadian canola cultivar. The admixtures included ECD 06–09 and two rutabagas.

### NJ and UPGMA cluster analyses

The NJ and UPGMA clustering was performed using 4,972 A-genome SNP markers for 194 *B. rapa* (45) and *B. napus* (149) genotypes, and 4,621 C-genome SNP markers for 166 *B. oleracea* (17) and *B. napus* (149) genotypes.

**Figure 4:**
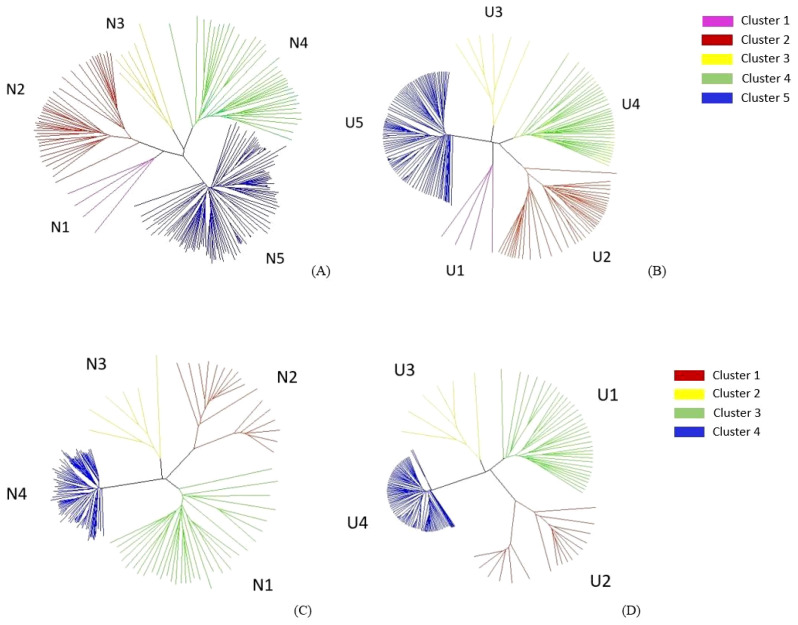
Neighbor joining (NJ) **(A)** and unweighted pair group method with arithmetic mean (UPGMA) **(B)** analysis with 4,972 A-genome markers grouped 194 *Brassica rapa* and *Brasscia napus* genotypes into five clusters. NJ **(C)** and UPGMA **(D)** analysis with 4,621 C-genome markers grouped 166 *Brassica oleracea* and *B. napus* genotypes into four clusters.

The cluster analyses of *B. rapa* and *B. napus*, using both the NJ and UPGMA methods, grouped them into five major branches. These comprised 4, 40, 34, 6, and 110 accessions in clusters 1, 2, 3, 4, and 5, respectively (**Figures 4A, B**). Cluster 1 (N1 and U1) included ECD 01–04 (*B. rapa*). Cluster 2 (N2 and U2) included 39 *B. rapa* vegetable cultivars and ECD 05. Cluster 3 (N3 and U3) encompassed ECD 06–09, two rutabagas, and one Canadian canola cultivar. Cluster 4 (N4 and U4) consisted of 34 Canadian canola cultivars and one *B. rapa* vegetable cultivar. The remaining 108 rutabagas, along with ECD 10 and one Canadian canola cultivar, were grouped into cluster 5 (N5 and U5) (**Figures 4A, B**).

In the *B. oleracea* and *B. napus* cluster analyses, both the NJ and UPGMA methods grouped the genotypes into four major clusters, where clusters 1, 2, 3, and 4 comprised 33, 17, 6, and 110 accessions, respectively (**Figures 4C, D**). Cluster 1 (N1 and U1) consisted of 33 Canadian canola cultivars. Cluster 2 (N2 and U2) included 15 *B. oleracea* vegetable cultivars, ECD 11, and ECD 13. Cluster 3 (N3 and U3) comprised ECD 06–09, one Canadian canola cultivar, and two rutabagas. The remaining 108 rutabagas, along with ECD 10 and one Canadian canola cultivar, were grouped as cluster 4 (**Figures 4C, D**).

### SNP-based association mapping of Verticillium stripe resistance loci

**Figure 5:**
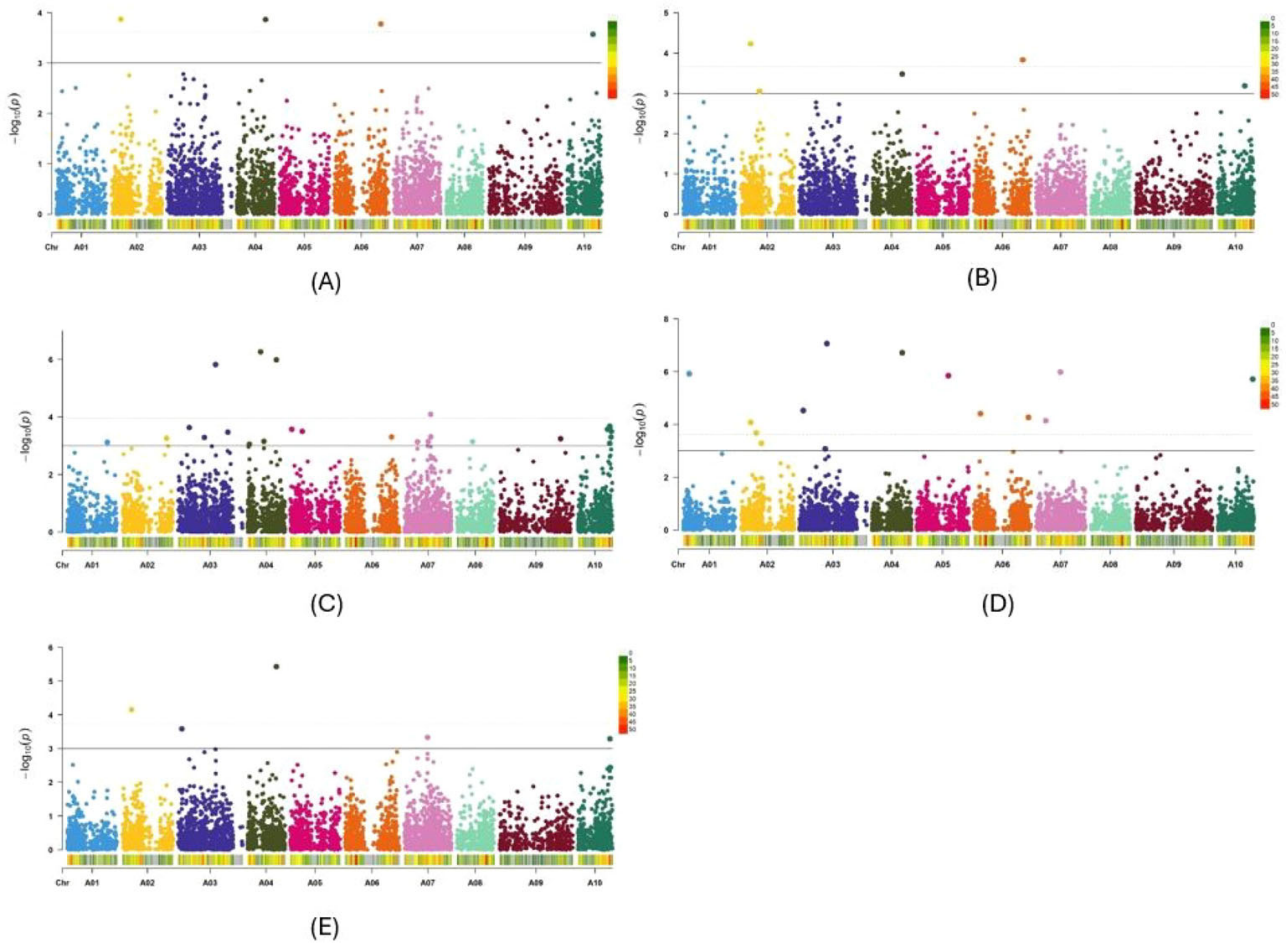
Manhattan plots of the PCA+K **(A)**, Q+K **(B)**, BLINK **(C)**, FarmCPU **(D)** and MLMM **(E)** models for identifying *Verticillium longisporum* resistance in *Brassica rapa* + *Brassica napus* genotypes. The dashed horizontal lines indicate the Bonferroni-adjusted significance threshold known as “logarithm-of-odds” (LOD score). The solid lines indicate a slightly lower threshold of -log10 *P* = 3.0. The dots above the significance threshold indicate single-nucleotide polymorphisms (SNPs) associated with resistance to *V. longisporum*.

**Figure 6:**
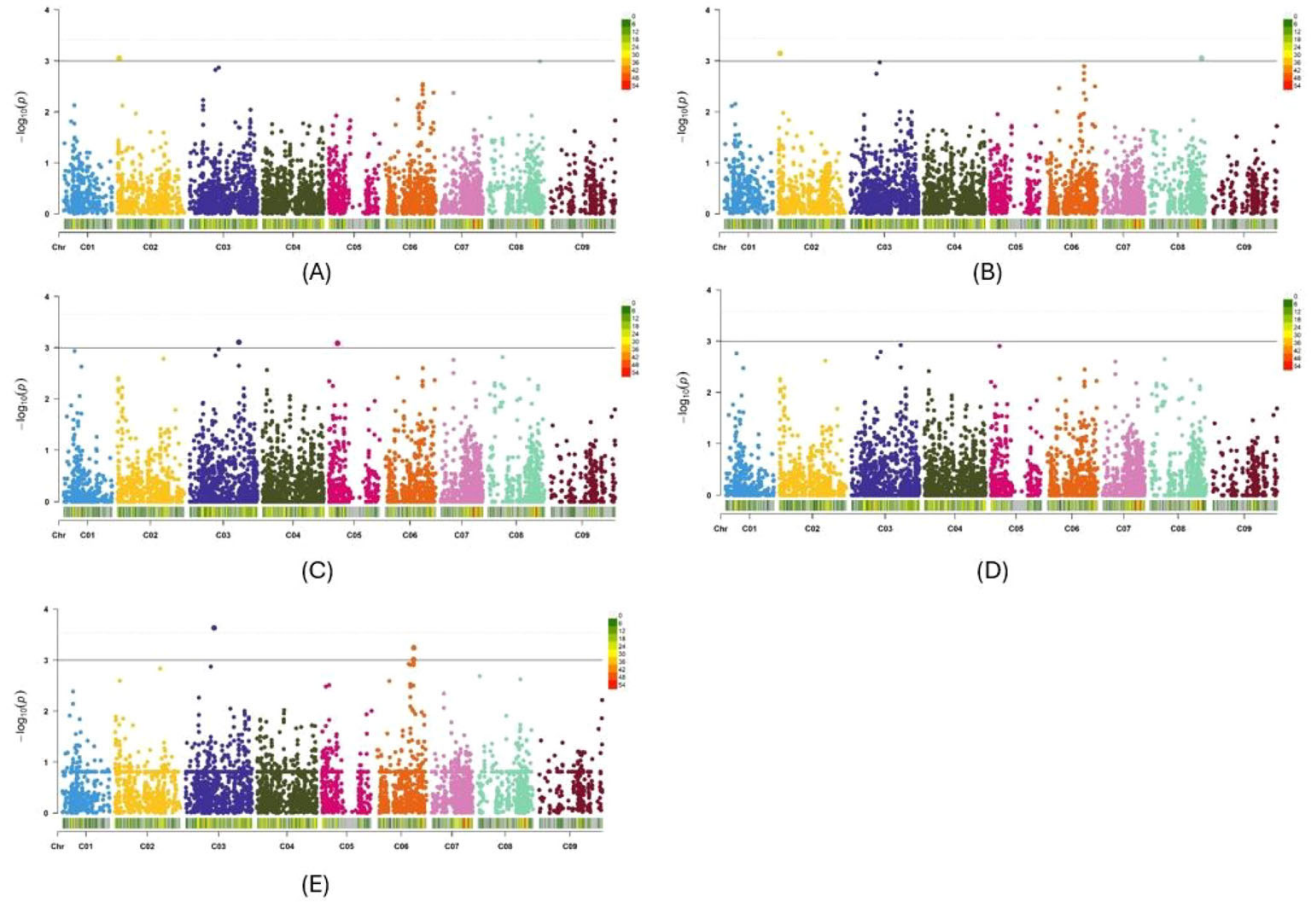
Manhattan plots of the PCA+K **(A)**, Q+K **(B)**, BLINK **(C)**, FarmCPU **(D)** and MLMM **(E)** models for identifying *Verticillium longisporum* resistance in *Brassica oleracea* + *Brassica napus* genotypes. The dashed horizontal lines indicate the Bonferroni-adjusted significance threshold known as “logarithm-of-odds” (LOD score). The solid lines indicate a slightly lower threshold of -log10 *P* = 3.0. The dots above the significance threshold indicate single-nucleotide polymorphisms (SNPs) associated with resistance to *V. longisporum*.

In the two GWAS, the observed -log_10_ *P* distribution showed greater deviation from the expected distribution in the Q-Q plots of the three GLMs than in the four MLMs. Among the four MLMs, the observed -log_10_ *P* distribution of the PCA + K and Q + K models deviated the least from the expected distribution compared with the Q + D and PCA + D models (**Supplementary Figures S3A–G, K–Q**). The observed -log_10_ *P* distribution of three other GWAS methods, namely BLINK, FarmCPU and MMLM, also exhibited minimal deviation from the expected distribution (**Supplementary Figures S3H–J, R–T**). Therefore, among the 10 models and methods tested, the PCA + K and Q+K models, along with the BLINK, FarmCPU, and MLMM methods, generated the best Q-Q plots. Consequently, Manhattan plots for these five methods were utilized to identify significant SNPs for Verticillium stripe resistance (**Figures 5A–E**, **6A–E**). Based on the Manhattan plots, 45 SNP markers were found to be associated with resistance to this disease (**Table 2**). Among these significant markers, 38 SNPs were identified on the A-genome, while seven SNPs were on the C-genome (**Table 2**). The significant SNPs were distributed across all chromosomes except for chromosomes C01, C04, C07, and C09 (**Table 2**). Markers identified on chromosome A and chromosome C explained 1.7% to 34.2% and 7.7% to 58.2% of variation, respectively (**Table 2**). Marker Bn_A03_p2130281 on chromosome A03 and Bn_scaff_18181_1_p572911 on chromosome C05 explained the highest percentage of variation of 34.2% and 58.2% respectively (**Table 2**). The markers effect size ranged from -0.33 to 0.35 and -0.19 to 0.43 for chromosome A and chromosome C, respectively (**Table 2**). The allelic effects of 45 SNP markers were listed in **Supplementary Table S2**.

**Table 2:** Single nucleotide polymorphism (SNP) markers identified in two genotype combinations, *Brassica rapa* + *Brassica napus* and *Brassica oleracea* + *B. napus*, including their chromosomal locations and linkage association with resistance to *Verticillium longisporum*.

Model Used^Θ^	^α^SNP marker	Da-Ae anchored, position of markers within LD blocks in bp	CAAD_Brap_v3.01 or BOL anchored, position of markers within LD blocks in bp	Marker R^2^	Effect
BLINK	Bn_A01_p21776155	chrA01:24428297–24428417	chrA01:23248025–23248145	5.9	0.17
FarmCPU	Bn_A01_p3134159	chrA01:3301020–3381218	chrA01:3198517–3278717	16.0	-0.23
Q+K	Bn_A02_p11087388	chrA02:9440260–9616460	chrA02: 10497328–10673528	6.3	-0.05
FarmCPU	Bn_A02_p12044265	chrA02: 10570943–10967143	chrA02: 11403200–11799352	4.6	0.15
BLINK	Bn_A02_p26154897	N/A	chrA02: 29935426–30277640	6.8	-0.20
FarmCPU	Bn_A02_p9353942	chrA02:7561609–7601809	chrA02:8672761–8712961	9.5	-0.13
FarmCPU/MLMM/PCA+K/Q+K	Bn_scaff_16269_1_p296261	chrA02:4394855–4427055	chrA02:5676675–5708875	4.9–12.4	0.13–0.22
FarmCPU	Bn_A03_p14037892	chrA03:14580053–14630353	chrA03:14958270–15008570	1.7	-0.11
BLINK/FarmCPU	Bn_A03_p14870270	chrA03:15469870–15470070	chrA03:15822618–15822618	7.2–13.5	-0.17- -0.15
FarmCPU/MLMM	Bn_A03_p2130281	chrA03:2006998–2111125	chrA03:2107003–2211123	32.5–34.2	0.20–0.29
BLINK	Bn_A03_p21487106	chrA03:21930598–22290718	chrA03:22425344–22785464	10.0	-0.24
BLINK	Bn_A03_p28202050	chrA03:29300983–29383078	chrA03:29592287–29674382	8.2	0.15
BLINK	Bn_A03_p6335597	chrA03:6428515–6442714	chrA03:6538542–6552741	8.7	0.21
BLINK	Bn_A04_p1311487	chrA04:1297104–1339403	chrA04:1434478–1476778	6.8	0.15
BLINK/FarmCPU/MLMM/PCA+K/Q+K	Bn_A04_p14410667	chrA04:16938776–17194896	chrA04:16952018–17208138	8.0–29.9	-0.33–0.35
BLINK	Bn_A04_p5853514	chrA04:8007251–8007450	chrA04:8099495–8099694	34.1	0.32
BLINK	Bn_A04_p7442886	chrA04:9831973–9866093	chrA04:9795253–9829373	2.3	-0.22
FarmCPU	Bn_A05_p14338060	chrA05:13729637–14685837	chrA05:16919715–17875915	2.5	0.15
BLINK	Bn_A05_p7098949	chrA05:7498441–7498560	chrA05:7526479–7526599	11.6	-0.19
BLINK	Bn_A05_p817036	chrA05:1132996–1133116	chrA05: 991522–991642	4.8	-0.26
BLINK/PCA+K/Q+K	Bn_A06_p22051862	chrA06:24378619–24496741	chrA06:24656692–24774812	8.1–15.8	-0.18- -0.16
FarmCPU	Bn_A06_p24886436	chrA06:27412672–27620972	chrA06:27628764–27837065	15.1	0.21
FarmCPU	Bn_A06_p3255819	chrA06:3482494–3818794	chrA06:3493071–3829371	20.4	-0.26
BLINK	Bn_A02_p771313	chrA07:16182323–16552623	chrA07:16933860–17304160	12.9	0.17
BLINK	Bn_A02_p808711	N/A	chrA07:16971615–17341735	7.9	-0.17
FarmCPU/MLMM	Bn_A07_p10370541	chrA07:14338605–14634725	chrA07:15154127–15450247	7.3–10.8	-0.20- -0.17
BLINK	Bn_A07_p3569093	chrA07:7584400–7780700	chrA07:8102342–8298648	16.3	0.16
FarmCPU	Bn_A07_p5030137	chrA07:9118996–9119116	chrA07:9697747–9697867	2.7	0.16
BLINK	Bn_scaff_18505_1_p254578	chrA07:14810135–14810335	chrA07:15605465–15605665	10.9	0.15
BLINK	Bn_A08_p6828854	chrA08:9828416–10804616	chrA08:9505318–10481518	5.1	0.18
BLINK	Bn_A09_p30329663	chrA09:37753992–37874111	chrA09:37892918–38013038	7.6	0.16
PCA+K/Q+K	Bn_A10_p15237975	chrA10:18521488–18521688	chrA10:18308874–18309041	6.9–7.6	-0.17- -0.14
BLINK/MLMM	Bn_A10_p15719803	chrA10:18026390–18050510	chrA10:18783483–18807603	22.1–26.4	-0.30- -0.28
BLINK	Bn_A10_p15727608	chrA10:17923152–18141270	chrA10:18693556–18911676	27.1	-0.28
BLINK	Bn_A10_p15731773	chrA10:17918089–18136209	chrA10:18697721–18915841	5.8	-0.29
BLINK	Bn_A10_p16620627	chrA10:18715856–18715973	chrA10:19718464–19718582	12.8	-0.16
BLINK/FarmCPU	Bn_A10_p16836688	chrA10:18845969–19018148	chrA10:19849511–20021711	9.3–20.7	-0.17- -0.16
BLINK	Bn_A10_p17367157	N/A	chrA10:20306024–20688144	15.0	-0.33
PCA+K/Q+K	Bn_scaff_15714_1_p2995346	chrC02:1142232–1458335	chrC02:1605204–1921404	8.8–9.0	0.10–0.11
MLMM	Bn_scaff_18482_1_p138097	chrC03:23145208–23233408	chrC03:22000155–22088355	19.5	-0.22
BLINK	Bn_scaff_19310_1_p376747	chrC03:39874412–40128532	chrC03:36831650–37085770	17.9	0.43
BLINK	Bn_scaff_18181_1_p572911	chrC05:8140181–8314481	chrC05:7563901–7738201	58.2	0.36
MLMM	Bn_scaff_15892_1_p310757	chrC06:35802282–36382402	chrC06:28824983–29405103	23.8	-0.18
MLMM	Bn_scaff_15892_1_p404259	chrC06:35884637–36464757	chrC06:28918485–29498605	16.4	-0.19
Q+K	Bn_A09_p33459299	N/A	chrC08:46230681–46230802	7.7	0.19

^Θ^Mixed Linear Model (MLM) designations: PCA, principal component analysis; Q, population structure; K, Kinship. GWAS method designations: BLINK, Bayesian-information and Linkage-disequilibrium Iteratively Nested Keyway; FarmCPU, the Fixed and random model Circulating Probability Unification; MLMM, the Multiple Locus Mixed Linear Model. ^α^SNP markers denoted with the same superscript letter mapped to multiple chromosomes on the reference genomes. The type of PCR-based markers showing trait association has been specified. Putative functions are based on matching entries in the EnsemblPlants and NCBI GenBank databases.

### Haplotype associated with verticillium resistance

The 9,593 (4,972 A-genome + 4,621 C-genome) SNP markers formed a mean of 2283 (1345 A-genome + 938 C-genome) blocks in the haplotype analyses. The haplotypes from the A-genome contained between 2 and 10 SNPs while the mean size of the haplotype blocks ranged from 47 to 90 kilobases (mean 65 kb). In the case of the C-genome, the number of SNPs in the haplotypes ranged from 2 to 24 while the mean size of the haplotype blocks ranged from 109 to 207 kb (mean 152 kb). The SS, FGR and CI genome association analyses yielded 25, 23 and 9 haplotype blocks, that were significantly associated with *V. longisporum* resistance, respectively (**Table 3**). Therefore, the SS and FGR methods significantly outperformed the CI method in identifying haplotype blocks. Twenty of the haplotype blocks were detected by both the SS and FGR methods whiles the SS and FGR methods independently detected 5 and 3 haplotype blocks, respectively (**Table 3**). Nine haplotype blocks were identified by the three methods. Altogether, a total of 28 haplotype blocks determined by the three methods (SS, GR FGR and CI) were used in the BlastN searches to determine the structural and functional genomic information. Our results showed that the haplotype-based GWAS analysis allowed the detection of more candidate genes than if only the SNP-based GWAS had been used. The haplotype-trait associations were located on 13 different chromosomes, with the LOD scores ranging from 1.8 to 69.7 (**Table 3**).

**Table 3:** Determination of haplotype blocks associated with Verticillium stripe resistance in canola determined using the confidence interval, four gamete rule, and solid spine of LD methods.

Haplotypes (significantly associated SNP)	Chrom	Confidence Interval	Four Gamete Rule	Solid Spine of LD
*hap*VLA1.1 (Bn_A01_p3134159)	A01	2 kb LD block 11	62 kb LD block 27	62 kb LD block 33
*hap*VLA2.1 (Bn_A02_p11087388)	A02	Unblocked SNP	Unblocked SNP	12 kb LD block 64
*hap*VLA2.2 (Bn_A02_p12044265)	A02	Unblocked SNP	70 kb LD block 70	69 kb LD block 74
*hap*VLA2.3 (Bn_scaff_16269_1_p296261)	A02	Unblocked SNP	16 kb LD block 26	Unblocked SNP
*hap*VLA3.1 (Bn_A03_p14037892)	A03	Unblocked SNP	25 kb LD block 80	Unblocked SNP
*hap*VLA3.2 (Bn_A03_p2130281)	A03	Unblocked SNP	45 kb LD block 10	21 kb LD block 14
*hap*VLA3.3 (Bn_A03_p21487106)	A03	Unblocked SNP	39 kb LD block 115	136 kb LD block 146
*hap*VLA3.4 (Bn_A03_p28202050)	A03	Unblocked SNP	Unblocked SNP	41 kb LD block 201
*hap*VLA3.5 (Bn_A03_p6335597)	A03	Unblocked SNP	70 kb LD block 27	Unblocked SNP
*hap*VLA4.1 (Bn_A04_p1311487)	A04	16 kb LD block 4	16 kb LD block 11	21 kb LD block 13
*hap*VLA4.2 (Bn_A04_p14410667)	A04	Unblocked SNP	57 kb LD block 92	64 kb LD block 103
*hap*VLA4.3 (Bn_A04_p7442886)	A04	15 kb LD block 18	51 kb LD block 48	51 kb LD block 56
*hap*VLA5.1 (Bn_A05_p14338060)	A05	Unblocked SNP	1 kb LD block 69	478 kb LD block 77
*hap*VLA6.1 (Bn_A06_p24886436)	A06	Unblocked SNP	73 kb LD block 113	458 kb LD block 151
*hap*VLA6.2 (Bn_A06_p3255819)	A06	Unblocked SNP	35 kb LD block 19	168 kb LD block 27
*hap*VLA7.1 (Bn_A02_p771313)	A07	Unblocked SNP	42 kb LD block 100	74 kb LD block 103
*hap*VLA7.2 (Bn_A02_p808711)	A07	2 kb LD block 45	2 kb LD block 101	109 kb LD block 104
*hap*VLA7.3 (Bn_A07_p10370541)	A07	23 kb LD block 39	23 kb LD block 86	23 kb LD block 86
*hap*VLA7.4 (Bn_A07_p3569093)	A07	0 kb LD block 24	98 kb LD block 46	98 kb LD block 44
*hap*VLA7.5 (Bn_scaff_18505_1_p254578)	A07	Unblocked SNP	Unblocked SNP	0 kb LD block 89
*hap*VLA8.1 (Bn_A08_p6828854)	A08	0 kb LD block 11	388 kb LD block 25	488 kb LD block 22
*hap*VLA10.1 (Bn_A10_p15719803)	A10	Unblocked SNP	15 kb LD block 86	10 kb LD block 112
*hap*VLA10.1 (Bn_A10_p15727608)	A10	Unblocked SNP	15 kb LD block 86	10 kb LD block 112
*hap*VLA10.1 (Bn_A10_p15731773)	A10	Unblocked SNP	15 kb LD block 86	61 kb LD block 111
*hap*VLA10.2 (Bn_A10_p17367157)	A10	Unblocked SNP	110 kb LD block 71	110 kb LD block 93
*hap*VLC2.1 (Bn_scaff_15714_1_p2995346)	C02	Unblocked SNP	158 kb LD block 5	158 kb LD block 6
*hap*VLC3.1 (Bn_scaff_18482_1_p138097)	C03	Unblocked SNP	Unblocked SNP	44 kb LD block 75
*hap*VLC3.2 (Bn_scaff_19310_1_p376747)	C03	57 kb LD block 64	57 kb LD block 123	57 kb LD block 119
*hap*VLC5.1 (Bn_scaff_18181_1_p572911)	C05	Unblocked SNP	Unblocked SNP	20 kb LD block 38
*hap*VLC6.1 (Bn_scaff_15892_1_p310757)	C06	318 kb LD block 48	318 kb LD block 60	319 kb LD block 60
*hap*VLC6.1 (Bn_scaff_15892_1_p404259)	C06	318 kb LD block 48	318 kb LD block 60	319 kb LD block 60

### Candidate genes based on SNP markers significantly associated with VL resistance

Based on the significant 38 SNP markers (**Table 2**) and 23 haplotype blocks on the A-genome (**Table 3**), a total of 962 genes on Da-Ae anchored genome assembly and 1143 genes on CAAS_Brap_v3.01 anchored genome assembly (**Supplementary Table S3**). Similarly, a total of 261 genes on Da-Ae anchored genome assembly and 242 genes (**Supplementary Table S3**) on BOL anchored genome assembly were detected for 7 SNP markers (**Table 2**) and 5 haplotype blocks (**Table 3**) identified on chromosome C. Among total of 2608 candidate genes, some of genes encode proteins such as disease resistance protein RML 1B-like, multisubstrate pseudouridine synthase 7, leucine-rich repeat receptor-like serine, L-type lectin-domain containing receptor kinase S.5, F-box protein family, ethylene-responsive transcription factor ERF106-like and serine/threonine-protein kinase 16-like that were reported to be associated with plant disease response (**Supplementary Table S3**). Other genes encoded functional proteins include formin-like protein, NAD-dependent protein, ATPase 1, plasma membrane-type-like, internal metabolism, and biosynthesis, which are associated with cellular and biochemical processes (**Supplementary Table S3**). Additionally, genes that encoded zinc transporter 12, COP1-interactive protein 1, transcription factor PIF3, and transcription factor DIVARICATA, play an important role in basic plant biological and physiological processes (**Supplementary Table S3**). Proteins of unknown molecular function were also detected (**Supplementary Table S3**).

## Discussion

As an emerging canola disease, Verticillium stripe continues to spread across the Canadian prairies (Oosterhuis, 2022), and has recently been detected in North Dakota (Chapara et al., 2023). However, no *V. longisporum* resistant canola cultivars have been registered in Canada, resulting in increased yield losses (Norman, 2023). Therefore, the identification of germplasm for breeding resistant cultivars and identifying molecular makers tightly linked with *V. longisporum* resistance for marker-assisted selection is critical. Association mapping, based on linkage disequilibrium of markers with QTLs, is a powerful tool for marker-assisted selection, enabling the exploitation of variation in plant materials (Jestin et al., 2011). GWAS is one of the most popular approaches for association mapping, offering significant advantages over linkage analysis. It provides higher resolution, incorporates a greater number of alleles, and allows for the simultaneous analysis of various traits of interest (Zhu et al., 2008). Currently, single nucleotide polymorphism (SNP) markers are widely utilized for GWAS (Ben-Ari and Lavi, 2012). These markers are co-dominant and suitable for high-throughput genotyping (Ben-Ari and Lavi, 2012). Their biallelic and high heritability contribute to increased genotyping accuracy (Zhu et al., 2008). In this study, GWAS was employed to find significant SNP markers associated with *V. longisporum* resistance in a large collection of *B. napus*, *B. rapa*, and *B. oleracea* genotypes.

The *Brassica* 15K SNP array used by Fredua-Agyeman et al. (2020) had a total of 13,714 SNP markers which comprised 7,214 A-genome markers and 6,500 C-genome markers which were mostly on scaffolds, while the 19K array had 9,966 A-genome markers and 8,886 C-genome markers which were mostly mapped to specific chromosomes. To minimize missing data, the *B. rapa* and *B. napus* accessions were analyzed separately using only the A-genome markers, while the *B. oleracea* and *B. napus* accessions were analyzed separately using only the C-genome markers. The filtered set of 4,972 SNP markers obtained from the *Brassica* 19K SNP array were uniformly distributed on the A_1_-A_10_ chromosomes and covered 291.6 Mb of the A-genome of *B. rapa* and *B. napus*. For the GWAS of the *B. oleracea* and *B. napus* accessions, the filtered set of 4,621 SNPs were also uniformly distributed on the C_1_-C_9_ chromosomes and covered 463.4 Mb of the C-genome. The two GWAS conducted in this study had approximately the same A-genome coverage and close to 1.5× more coverage of the C-genome compared to a previous study that utilized a *Brassica* 15K SNP array from SGS TraitGenetics (Fredua-Agyeman et al., 2020). In the current study, the mean maker density was 61.6 ± 21.5 Kb on the A-genome, and 117.8 ± 59.3 Kb on the C-genome. In contrast, Fredua-Agyeman et al. (2020) reported mean marker densities using the *Brassica* 15K SNP assay of approximately 63.4 ± 21.9 Kb and 15.0 ± 8.4 Kb for the A- and C-genomes, respectively. Therefore, the marker density on the A-genome was similar, but on the C-genome, the density was almost 9× lower with the 19K SNP array used in this study. While the 19K SNP array provided increased coverage, more C-genome markers need to be developed.

Linkage disequilibrium refers to the association and linkage of alleles among SNPs within a genomic sequence, which is important in GWAS for identification of genetic markers (Joiret et al., 2022). Wang et al. (2017) observed that the extent of LD decay ranged from 0.15 to 3.3 Mb for the A-genome and from 0.4 to 8.3 Mb for the C-genome. Using a *Brassica* 60K Illumina Infinium SNP array, Xu et al. (2015), reported LD decay for the A-genome ranging from 0.6 to 5.6 Mb and from 1.2 to 8.5 Mb for the C-genome. In another study, Fredua-Agyeman et al. (2020) estimated LD decay ranging from 1.1 to 2.3 Mb for the A-genome and from 0.20 to 1.5 Mb for the C-genome using the *Brassica* 13.2K SNP array. The extent of LD decay found in this study ranged from 0.42 to 1.15 Mb for the A-genome and from 0.68 to 1.85 Mb for the C-genome, which was similar to the values reported in these earlier studies.

In the present study, GWAS was conducted using three GLMs (Q-only, K-only, and PCA-only) and four MLMs (PCA+D, PCA+K, Q+K, and Q+D), in addition to three methods (BLINK, FarmCPU, and MLMM). Mixed linear models are versatile and widely used in GWAS. They offer a balance between complexity and computational efficiency by incorporating population structure and kinship to adjust associations on markers (Yang et al., 2014). However, MLMs can lead to increased false positive rates due to overfitting (Kaler et al., 2020). Therefore, other GWAS algorithms were also employed in this study. To reduce false positive or false negative associations, BLINK considers both main effects and interactions among genetic variants (Huang et al., 2019). FarmCPU integrates fixed and random effects and adjusts for population structure and relatedness using a kinship matrix (Kaler et al., 2020), while MLMM simultaneously considers multiple loci, accommodating polygenic effects (Kaler et al., 2020). By employing multiple GWAS methods, SNP markers could be identified more consistently, thereby increasing the accuracy and efficiency of QTL detection.

In this study, SNP-based GWAS identified 45 significant SNP markers (38 were A-genome + 7 C-genome) to be associated with Verticillium stripe resistance. The distribution of the significant SNP markers were 2, 5, 6, 4, 3, 3, 6, 1, 1, 7, 1, 2, 1, 2 and 1 and these were found on chromosomes A01, A02, A03, A04, A05, A06, A07, A08, A09, A10, C02, C03, C05, C06 and C08, respectively. In comparison, haplotype-based GWAS identified 9 to 25 haploblocks. The use of haplotypes in GWAS provides additional information for genomic prediction, resulting in higher accuracies (Liu et al., 2019). Consequently, these significant SNP markers were further analyzed to identify haploblocks, which are associated with extended genomic regions containing candidate genes. Multiple markers identified within the same haploblock were considered to be within a single QTL region. In total, the 45 significant SNP markers and the 9 to 25 haploblocks identified 42 QTLs. These QTLs contained 1,223, 1,143, and 242 candidate genes when aligned to the genomes of *B. napus*, *B. rapa* and *B. oleracea*, respectively.

Six SNPs, each corresponding to a distinct QTL, were identified on chromosome A03 based on the SNP-based association and haplotype block analysis. Gabur et al. (2020) mapped the major QTLs for VL resistance to the A03 chromosome using a biparental DH population. In this GWAS study, the significant SNPs on A03 seem to be distal from the SNPs bordering the QTL for *V. longisporum* resistance previously reported by Gabur et al. (2020) on chromosome A03 (physical position 7,963,059 to 11,419,476). Therefore, the QTL regions on chromosome A03 identified in this study appear to be different from the QTLs reported by Gabur et al. (2020). One of the SNP markers identified in this study, Bn_A03_p14870270, was found in a genomic region reported to be a hotspot for clubroot resistance (Fredua-Agyeman et al., 2021). This region contains the clubroot resistance gene(s)/QTLs *Bn.A3P2F*, *Crr3*, *CRk*, and *CRd*. However, this SNP did not form a haploblock but was found to be in the genomic region encoding multisubstrate pseudouridine synthase. The haplotype *hap*VLA10.1 was associated with three significant SNP markers: Bn_A10_p15719803, Bn_A10_p15727608, and Bn_A10_p15731773. The haplotype *hap*VLC6.1 was associated with two significant SNP markers, Bn_scaff_15892_1_p310757 and Bn_scaff_15892_1_p404259.

The rutabaga accessions used in this study were also screened for clubroot resistance by Fredua-Agyeman et al. (2020). Unfortunately, accessions previously classified as resistant or moderately resistant to clubroot (FGRA036, FGRA037, FGRA044, FGRA072, FGRA106, FGRA108, FGRA109 and FGRA112) were found to be susceptible or moderately susceptible to *V. longisporum*. Hirani et al. (2018) reported that ECD 01, ECD 02, ECD 03 and ECD 04 (*B. rapa*), as well as ECD 11 (*B. oleracea*), were resistant to Canadian field isolates of the clubroot pathogen, while ECD 05 (*B. rapa*) was susceptible. In the current study, ECD 01, ECD 02, ECD 03 and ECD 04 were all susceptible to *V. longisporum* isolate VL 43. Among all of the ECD hosts screened, only ECD 11 exhibited resistance to this fungus, while ECD 05 was moderately resistant. Similarly, ECD 08 (*B. napus*) and ECD 13 (*B. oleracea*) were moderately resistant to *V. longisporum*, but they were susceptible and segregated in response to clubroot (Hirani et al., 2018). Consequently, the present findings suggest a negative relationship between resistance to Verticillium stripe and clubroot.

Of the 20 Verticillium stripe-resistant *Brassica* genotypes identified in this study, eight were *B. rapa* and *B. oleracea* vegetable types, three were rutabagas, and nine were canola. Gabur et al. (2020); Rygulla et al. (2007), and Su et al. (2023) identified QTLs/genes for Verticillium stripe resistance in vegetable-type *Brassica* germplasm. This indicates that *B. rapa* and *B. oleracea* might be potential donors for resistance breeding programs in canola/oilseed rape. Nine or about a quarter of the Canadian canola cultivars in this study were found to be resistant to *V. longisporum*, suggesting that the deployment of resistant hosts holds promise for the management of Verticillium stripe.

In the current GWAS, genes located within the QTLs were identified that were associated with plant disease resistance and immunity mechanisms. The gene identified in the QTL region on chromosome A04 encoded disease resistance protein RML1B-like. It was reported to be related to *Leptosphaeria maculans* resistance in *A. thaliana* (Staal et al., 2006). Similarly, genes encoding disease resistance protein RML1A-like on chromosome A07 were identified as involved in resistance to powdery mildew (Bhattarai et al., 2020). In one of the QTL regions on chromosome A06, the gene encoded disease resistance protein RPV1-like was identified which confers resistance to *Plasmopara viticola* (Feechan et al., 2013). In the other QTL region on chromosome A06, genes encoding disease resistance protein RBA1-like and TMV resistance protein N were identified, which were associated with cell death for disease resistance in Arabidopsis (Nishimura et al., 2017) and resistance to tobacco mosaic virus (Marathe et al., 2002). Additionally, the gene encoding protein PYRICULARIA ORYZAE RESISTANCE 21 was identified in the QTL region on chromosome A08 which confers resistance to *Magnaporthe oryzae* (Nawaz et al., 2020). Unlike QTL regions on the A-genome, only one of the regions on chromosome C08 identified a gene encoding a probable disease resistance protein At1g15890, which was reported as an *Arabidopsis*-resistance gene related to cell death (Huang et al., 2024). Other genes encoded proteins that were associated with disease resistance responses such as the ethylene-responsive transcription factor ERF106 (Yang et al., 2022), a probable leucine-rich repeat receptor-like protein kinase which modulates the development of *Phytophthora sojae* on soybean (Si et al., 2021), and an L-type lectin-domain containing receptor kinase that was found to positively regulate disease resistance against *Phytophthora* in pepper (Woo et al., 2020). It is possible that these QTL regions habour genes controlling *V. longisporum* resistance.

In conclusion, screening 211 *Brassica* genotypes for resistance to *V. longisporum* identified 20 resistant accessions/cultivars, including representatives from *B. rapa*, *B. oleracea*, and *B. napus*. Additionally, significant SNP markers on chromosome A03 may be important for Verticillium stripe resistance breeding.

## Data Availability

The original contributions presented in the study are included in the article/**Supplementary Material**. Further inquiries can be directed to the corresponding authors.
