# Supplementary material for: Genome-wide association study of Verticillium longisporum resistance in Brassica genotypes
Source: Front Plant Sci. 2024 Aug 27;15:1436982. doi: 10.3389/fpls.2024.1436982 (PMC11384582; doi:10.3389/fpls.2024.1436982)
Supplement: Supplementary file 2 [file DataSheet1.docx]

**Supplementary Figures**

(a)

**Supplementary Figure S1A.** *Verticillium longisporum* resistance responses measured by area under the disease progress curve (AUDPC_norm,_) for 110 rutabagas (*B. napus*) compared to the susceptible check ‘Westar’ (red) and moderately resistant check ‘Granaat’ (ECD 05) (green). Columns and bars represent mean values of four replicates of round 1, round 2 and average of two rounds with standard deviations. Round 1 represents the first independent repeat; round 2 represents the second independent repeat and average represents the mean AUDPC values of two independent repeats.

(b)

**Supplementary Figure S1B.** *Verticillium longisporum* resistance responses measured by area under the disease progress curve (AUDPC_norm,_) for 34 of the 35 Canadian canola cultivars (*B. napus*) compared to the susceptible check ‘Westar’ (red) and moderately resistant check ‘Granaat’ (ECD 05) (green). Columns and bars represent mean values of four replicates of round 1, round 2 and average of two rounds with standard deviations. Round 1 represents the first independent repeat; round 2 represents the second independent repeat and average represents the mean AUDPC values of two independent repeats.

(c)

**Supplementary Figure S1C.** *Verticillium longisporum* resistance responses measured by area under the disease progress curve (AUDPC_norm,_) for 40 *B. rapa* vegetable cultivars from China compared to the susceptible check ‘Westar’ (red) and moderately resistant check ‘Granaat’ (ECD 05) (green). Columns and bars represent mean values of four replicates of round 1, round 2 and average of two rounds with standard deviations. Round 1 represents the first independent repeat; round 2 represents the second independent repeat and average represents the mean AUDPC values of two independent repeats.

(d)

**Supplementary Figure S1D.** *Verticillium longisporum* resistance responses measured by area under the disease progress curve (AUDPC_norm,_) for 15 *B. oleracea* vegetable cultivars from China compared to the susceptible check ‘Westar’ (red) and moderately resistant check ‘Granaat’ (ECD 05) (green). Columns and bars represent mean values of four replicates of round 1, round 2 and average of two rounds with standard deviations. Round 1 represents the first independent repeat; round 2 represents the second independent repeat and average represents the mean AUDPC values of two independent repeats.

(e)

**Supplementary Figure S1E.** *Verticillium longisporum* resistance responses measured by area under the disease progress curve (AUDPC_norm,_) for 10 selected hosts of the ECD set apart from ECD05 (‘Granaat’) compared to the susceptible check ‘Westar’ (red) and moderately resistant check ‘Granaat’ (ECD 05) (green). Columns and bars represent mean values of four replicates of round 1, round 2 and average of two rounds with standard deviations. Round 1 represents the first independent repeat; round 2 represents the second independent repeat and average represents the mean AUDPC values of two independent repeats.


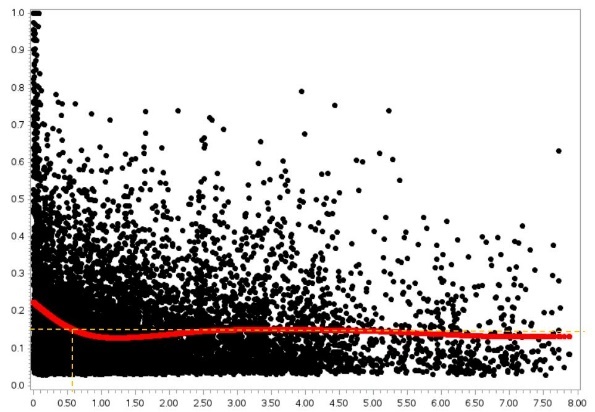
(A)
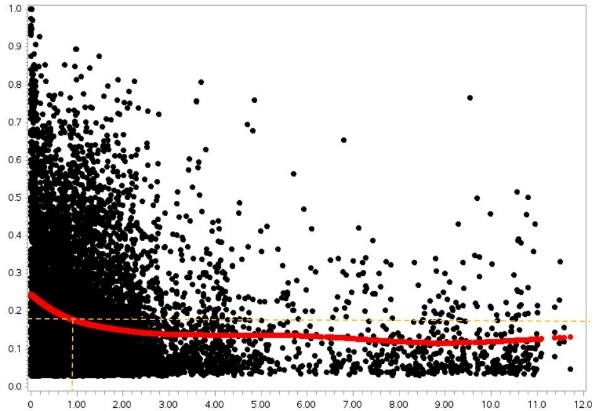
(B)
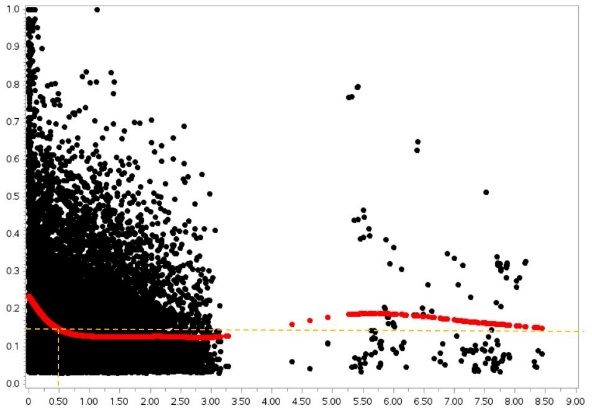
(C)
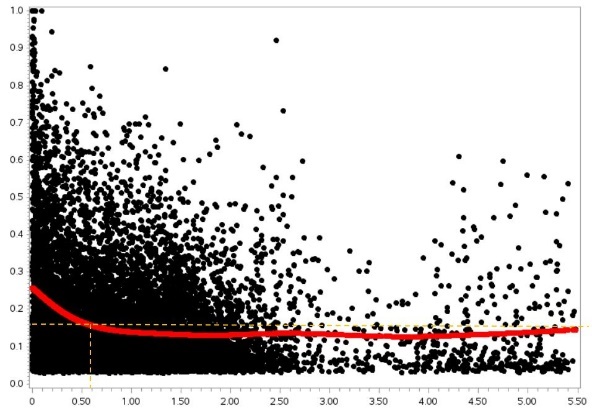
 (D)
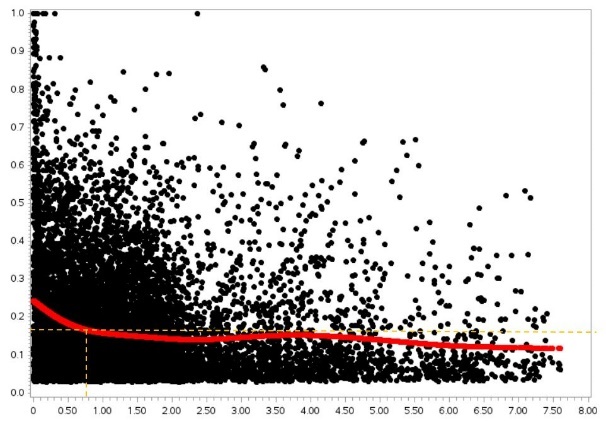
(E)
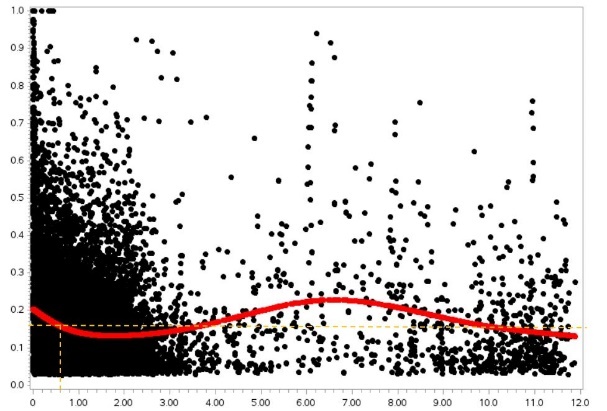
(F)


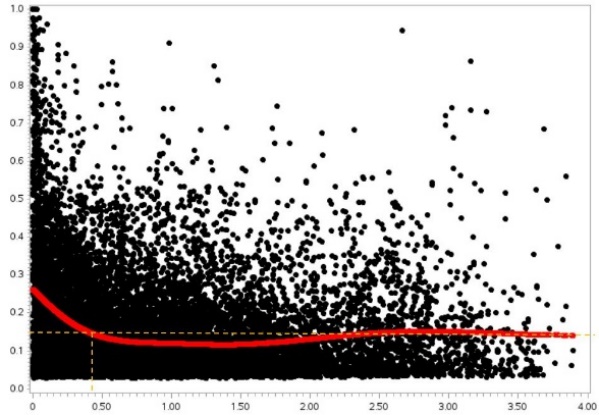
(G)
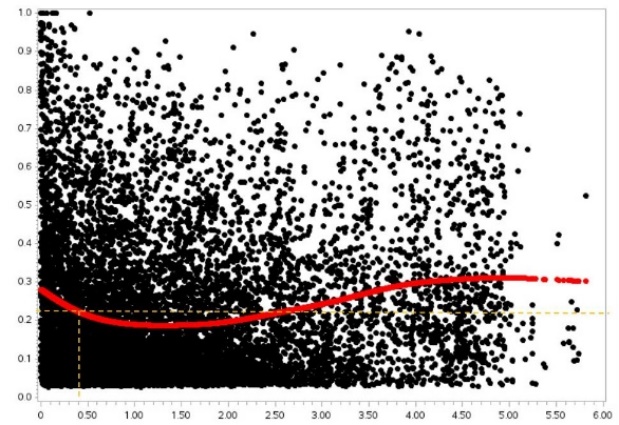
(H)
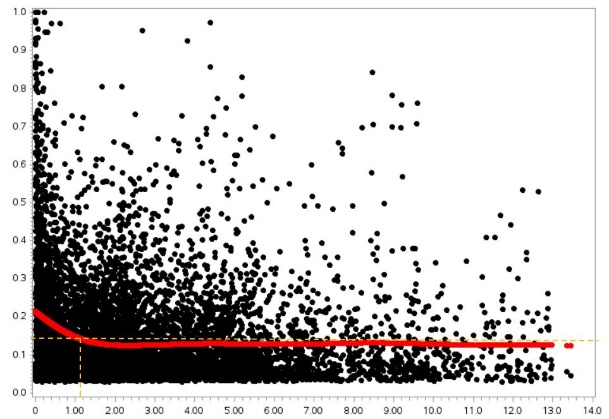
(I)


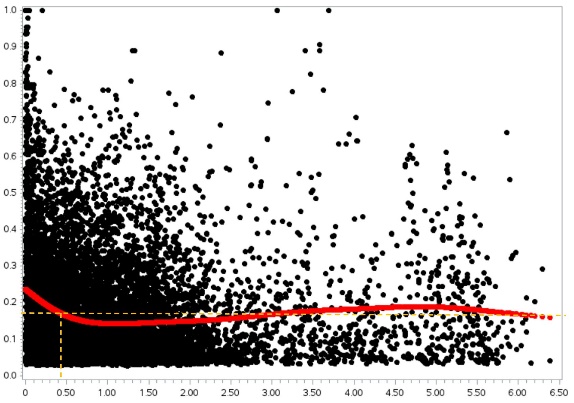
(J)
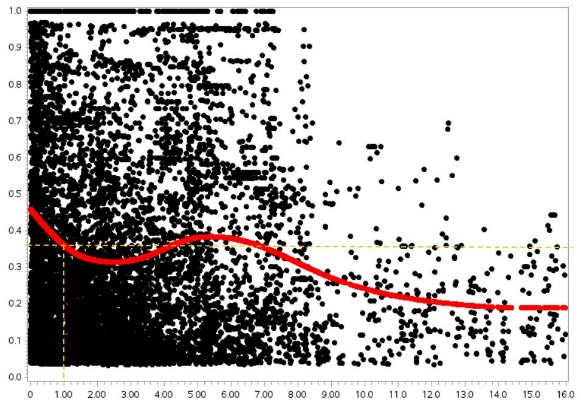
(K)
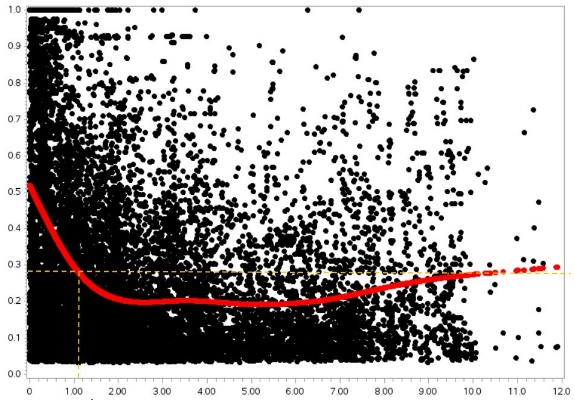
(L)
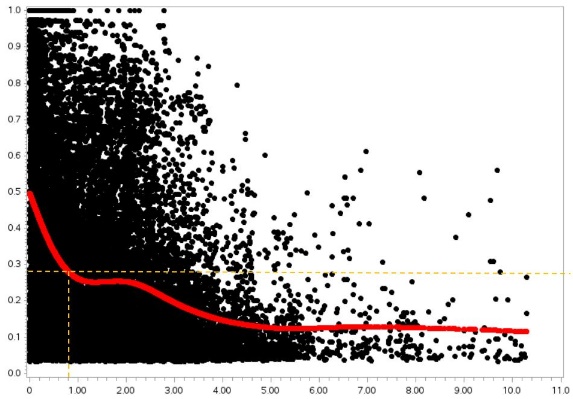
(M)
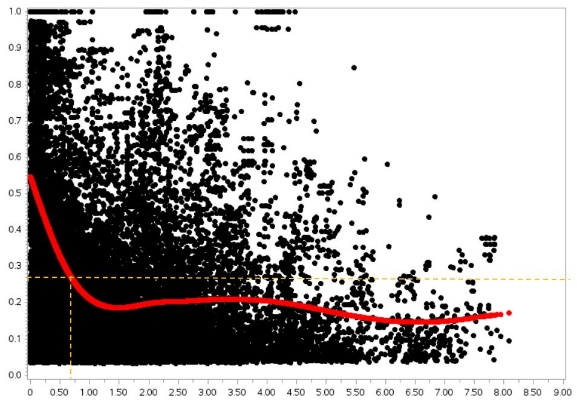
(N)
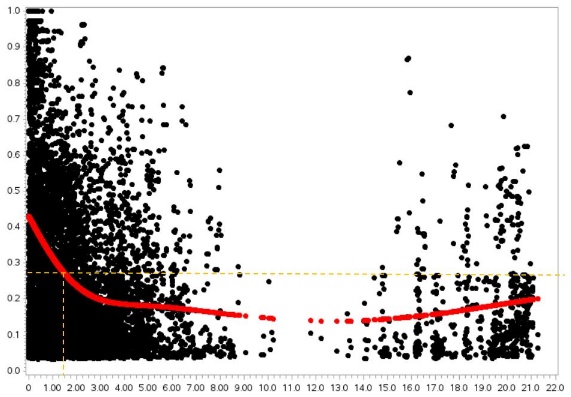
(O)
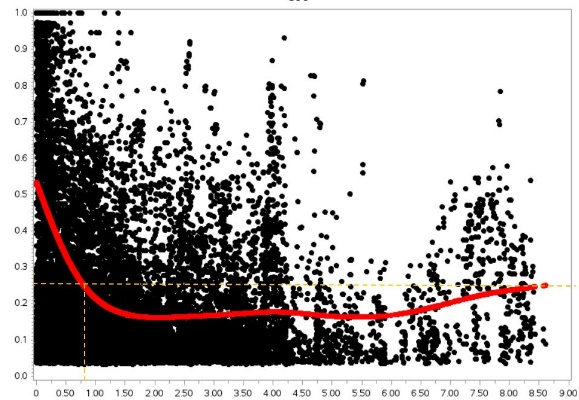
(P)
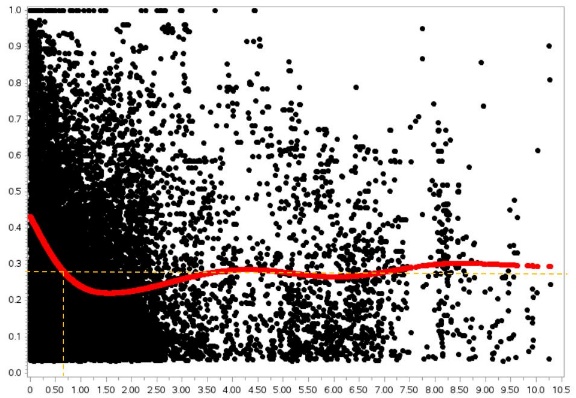
(Q)
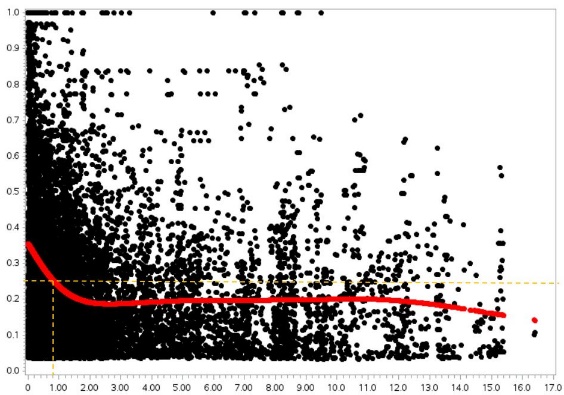
(R)
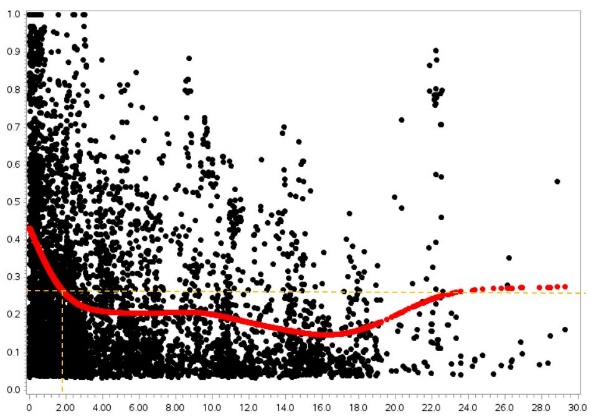
(S)

Supplementary Figure 2. Plots of correlation coefficient (r^2^) and physical distance (in Mb) for SNP markers on chromosomes A01 – A10 (A-J) and chromosomes C01 – C09 (K-S). The red curves represent the fitted plots of the data points, while the yellow line represents the background linkage disequilibrium (BLD) or threshold line. The decay of linkage disequilibrium was determined by projecting the intersection of the curves and the BLD line onto the physical distance axis.


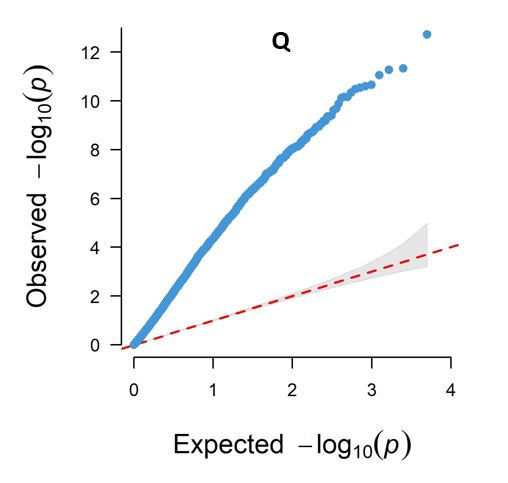
(A)
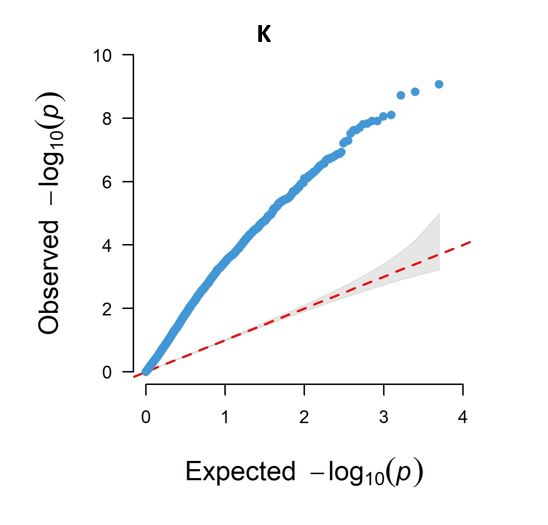
(B)


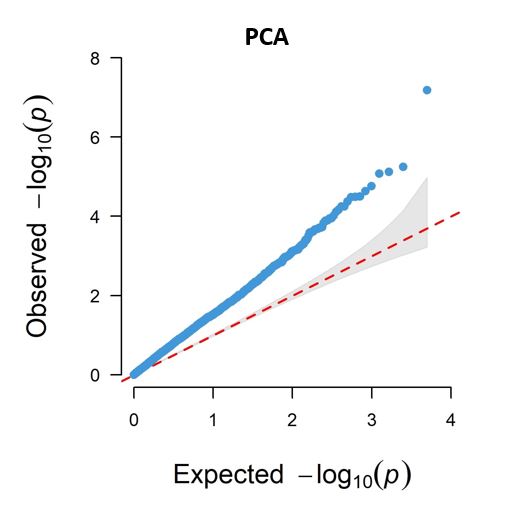
(C)
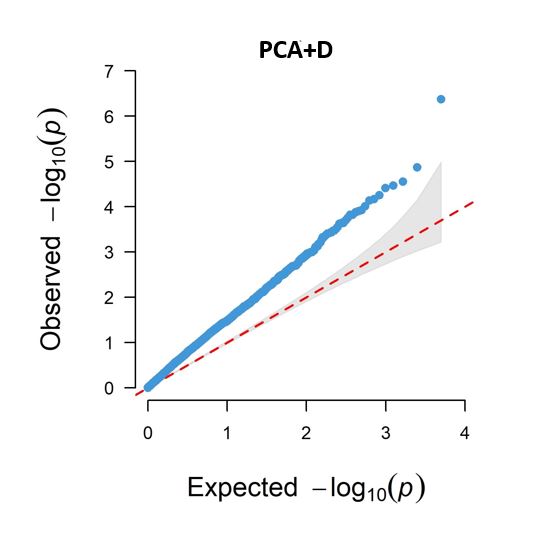
(D)


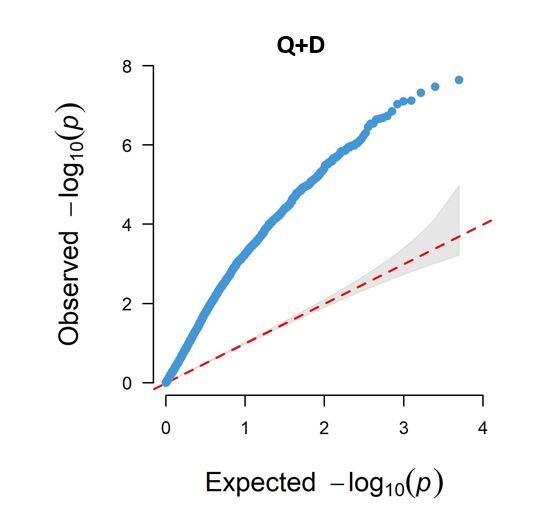
(E)
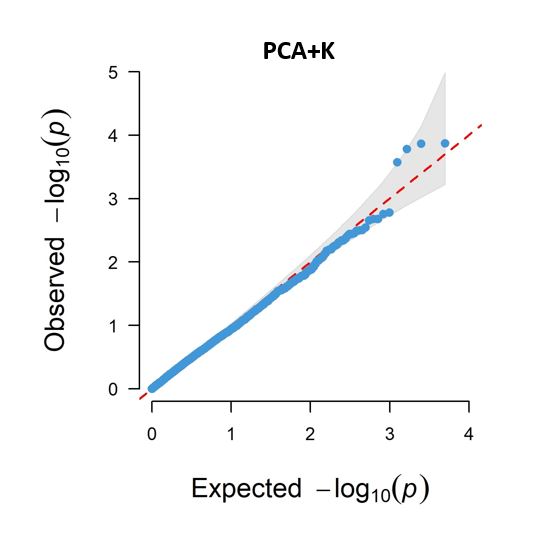
(F)


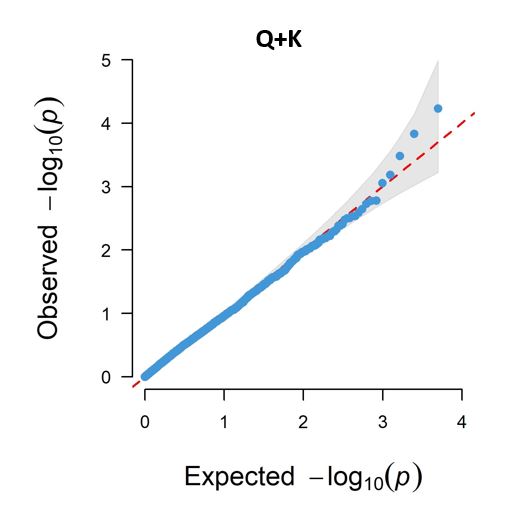
(G)
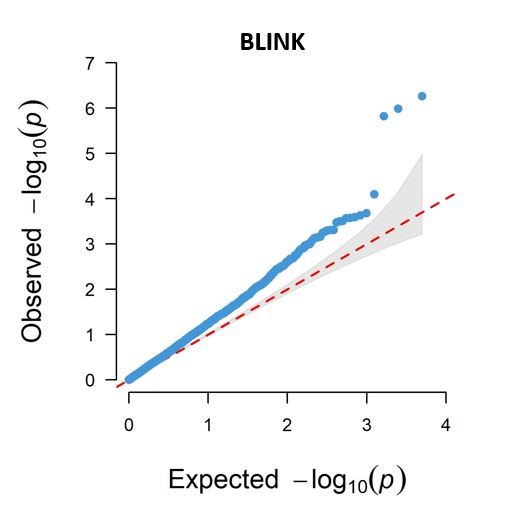
(H)


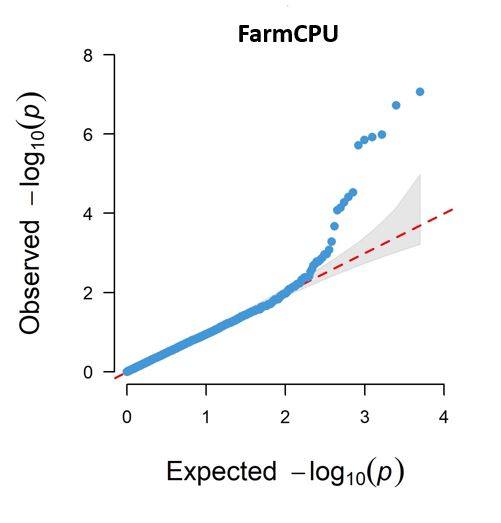
(I)
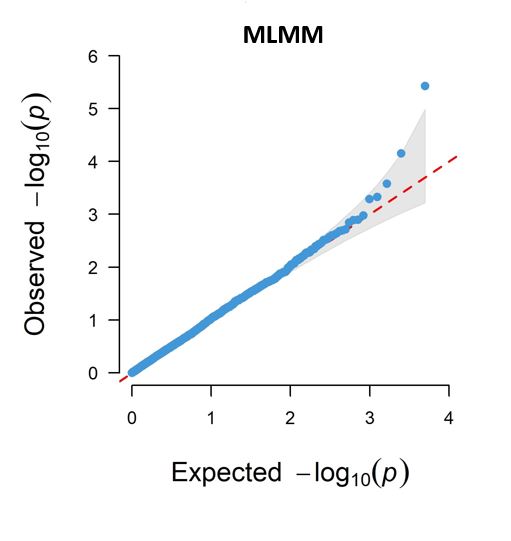
(J)


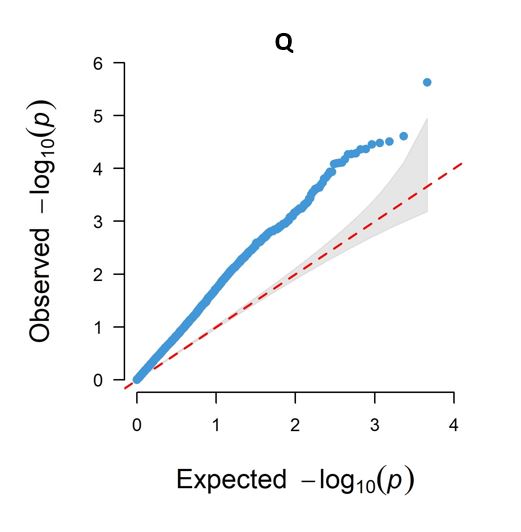
(K)
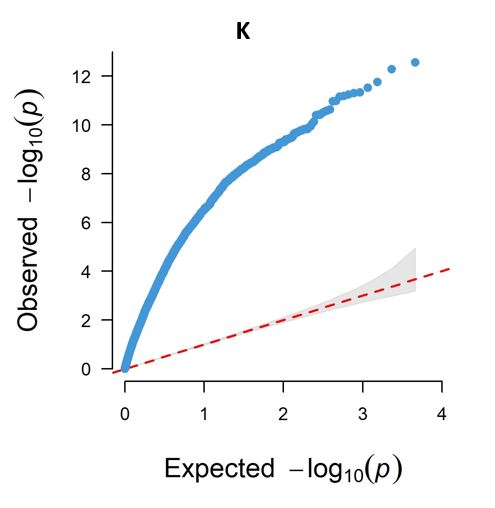
(L)


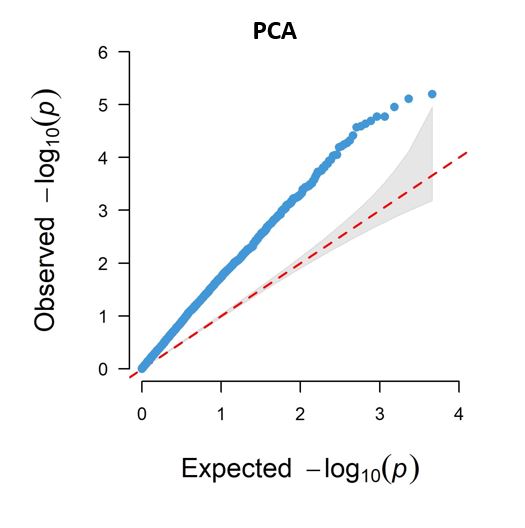
(M)
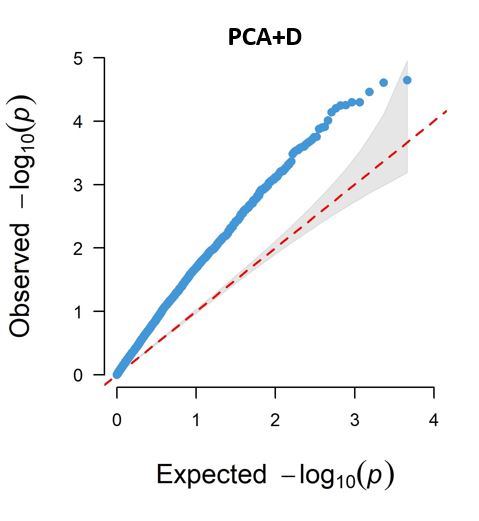
(N)


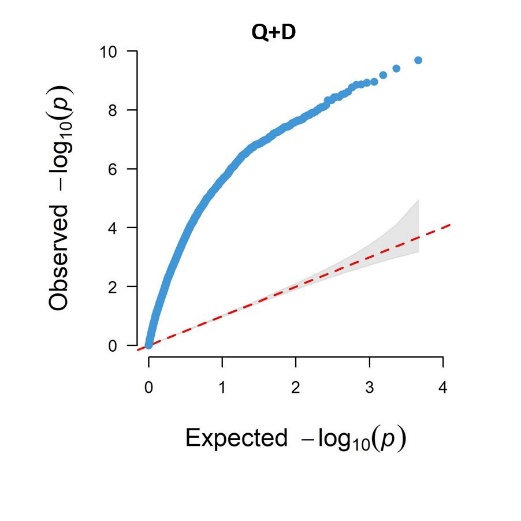
(O)
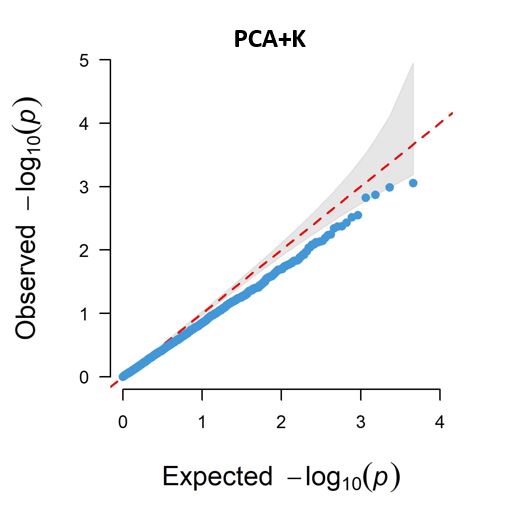
(P)


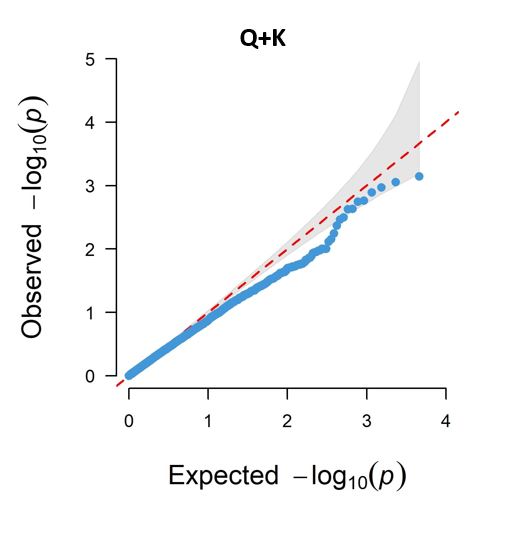
(Q)
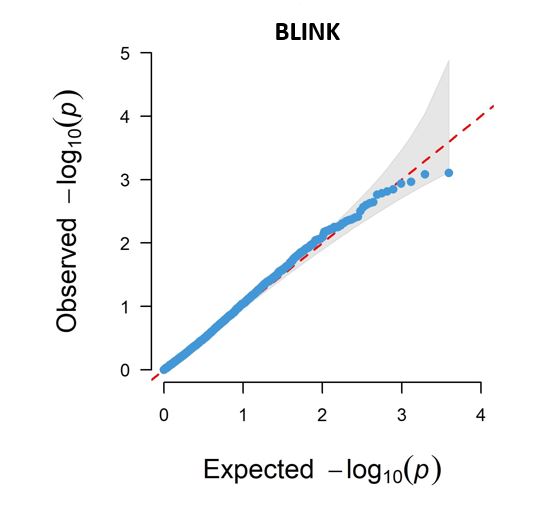
(R)


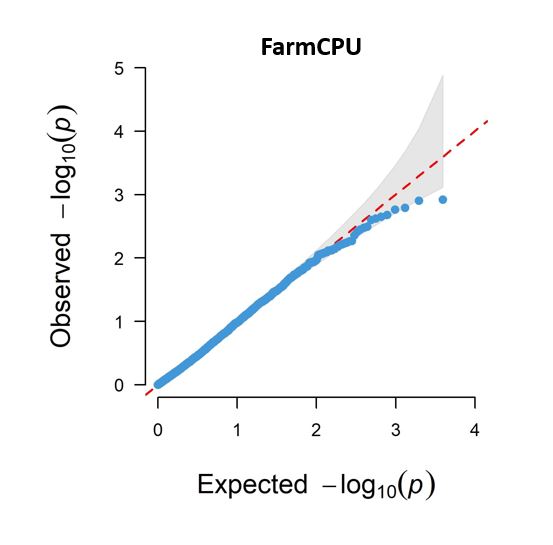
(S)
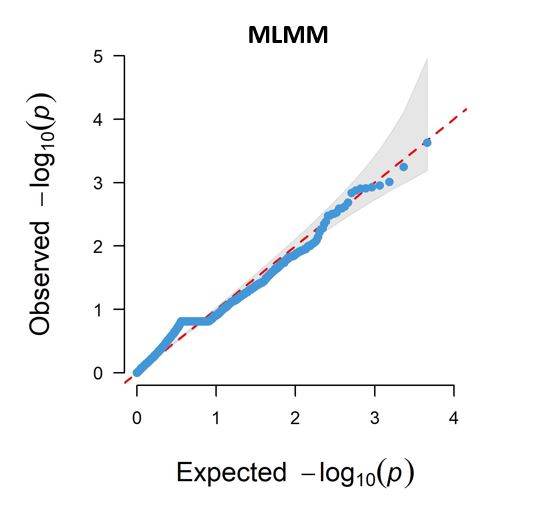
(T)

**Figure S3.** Quantile-Quantile comparison of GWAS model Q-only (A), K-only (B), PCA-only (C), PCA+D (D), Q+D (E), PCA+K (F), Q+K (G), BLINK (H), FarmCPU (I) and MLMM (J) models for *B. rapa* + *B. napus* genotypes. QQ-plot of GWAS model Q-only (K), K-only (L), PCA-only (M), PCA+D (N), Q+D (O), PCA+K (P), Q+K (Q), BLINK (R), FarmCPU (S) and MLMM (T) models for *B. oleracea* + *B. napus* genotypes. The red dash line is the expected -log10 *P* distribution while colored lines are the observed -log10 *P* distribution.
